# Comprehensive Adaptive Enterprise Optimization Algorithm and Its Engineering Applications

**DOI:** 10.3390/biomimetics10050302

**Published:** 2025-05-09

**Authors:** Shuxin Wang, Yejun Zheng, Li Cao, Mengji Xiong

**Affiliations:** 1 School of Intelligent Manufacturing, Shanghai Zhongqiao Vocational and Technical University, Shanghai 201514, China; 2 Engineering Technology Department, Shanghai Caoyang Vocational School, Wenzhou 200065, China; 3 School of Intelligent Manufacturing and Electronic Engineering, Wenzhou University of Technology, Wenzhou 325035, China

**Keywords:** enterprise optimization algorithm, engineering application, tent chaotic mapping, lens imaging strategy, reverse learning strategy

## Abstract

In this study, a brand-new algorithm called the Comprehensive Adaptive Enterprise Development Optimizer (CAED) is proposed to overcome the drawbacks of the Enterprise Development (ED) algorithm in complex optimization tasks. In particular, it aims to tackle the problems of slow convergence and low precision. To enhance the algorithm’s ability to break free from local optima, a lens imaging reverse learning approach is incorporated. This approach creates reverse solutions by utilizing the concepts of optical imaging. As a result, it expands the search range and boosts the probability of finding superior solutions beyond local optima. Moreover, an environmental sensitivity-driven adaptive inertial weight approach is developed. This approach dynamically modifies the equilibrium between global exploration, which enables the algorithm to search for new promising areas in the solution space, and local development, which is centered on refining the solutions close to the currently best-found areas. To evaluate the efficacy of the CAED, 23 benchmark functions from CEC2005 are chosen for testing. The performance of the CAED is contrasted with that of nine other algorithms, such as the Particle Swarm Optimization (PSO), Gray Wolf Optimization (GWO), and the Antlion Optimizer (AOA). Experimental findings show that for unimodal functions, the standard deviation of the CAED is almost 0, which reflects its high accuracy and stability. In the case of multimodal functions, the optimal value obtained by the CAED is notably better than those of other algorithms, further emphasizing its outstanding performance. The CAED algorithm is also applied to engineering optimization challenges, like the design of cantilever beams and three-bar trusses. For the cantilever beam problem, the optimal solution achieved by the CAED is 13.3925, with a standard deviation of merely 0.0098. For the three-bar truss problem, the optimal solution is 259.805047, and the standard deviation is an extremely small 1.11 × 10^−7^. These results are much better than those achieved by the traditional ED algorithm and the other comparative algorithms. Overall, through the coordinated implementation of multiple optimization strategies, the CAED algorithm exhibits high precision, strong robustness, and rapid convergence when searching in complex solution spaces. As such, it offers an efficient approach for solving various engineering optimization problems.

## 1. Introduction

In today’s modern world, optimization problems are ubiquitous across a diverse range of real-life systems. They are of vital importance in guaranteeing the efficient functioning and performance of these systems. For example, in the field of fault diagnosis, precisely determining the underlying causes of faults and optimizing the diagnostic procedure can result in prompt repairs and a reduction in downtime, thereby saving substantial costs for various industries. In energy management, optimizing the distribution and utilization of energy sources is fundamental for attaining sustainable development, minimizing environmental impacts, and increasing energy efficiency. Prediction, whether it involves forecasting market trends, natural disasters, or equipment malfunctions, necessitates advanced optimization methods to enhance the accuracy of predictions and enable proactive decision-making [1,2]. These real-world systems frequently pose complex optimization hurdles. They usually entail large-scale problems, where the solution space is extensive and difficult to thoroughly explore. Moreover, the existence of multiple extreme values makes it arduous to differentiate between local optima and the global optimum. Traditional mathematical programming techniques, like the conjugate gradient method and the quasi-Newton method, have been employed for a long time to solve optimization problems. Nevertheless, these methods depend on the gradient information of the objective function and make assumptions regarding the smoothness and convexity of the function. When confronted with complex real-world problems that have non-convex, discontinuous, or high-dimensional solution spaces, their performance is severely restricted. They often become stuck in local optima, are unable to converge to the global optimum, or demand excessive computational resources, rendering them ineffective in dealing with these challenging optimization tasks [3].

On the other hand, swarm intelligence optimization algorithms have emerged as a potent alternative. As a population-oriented evolutionary computing technology, they take inspiration from the collective behaviors of biological groups in the natural world, such as ants, birds, fish, and bees. These algorithms simulate the social interactions, communication, and cooperation among individuals within these groups to search for optimal solutions [4]. By maintaining a population of candidate solutions and evolving them over multiple generations through operations such as selection, crossover, and mutation, swarm intelligence algorithms can explore the solution space more comprehensively and avoid being trapped in local optima. They demonstrate greater adaptability to different problem characteristics and are capable of handling complex, nonlinear, and multimodal optimization problems [5].

There are several notable representatives of swarm intelligence optimization algorithms. The Particle Swarm Optimization algorithm (PSO) is inspired by the swarming behavior of birds or the schooling behavior of fish. In PSO, each particle represents a potential solution in the search space, and particles update their positions based on their own best-known position and the best-known position within the entire swarm. This straightforward yet effective mechanism enables PSO to rapidly converge to satisfactory solutions in numerous optimization problems [6]. The chicken swarm optimization algorithm (CSO) models the hierarchical social structure and foraging behavior of chickens. It divides the population into different subgroups, such as roosters, hens, and chicks, with each subgroup having its own behavioral rules. This hierarchical structure assists CSO in effectively balancing exploration and exploitation and enhancing the optimization performance [7]. The Gray Wolf Optimization algorithm (GWO) is inspired by the leadership hierarchy and hunting behavior of gray wolves in nature. It utilizes the concepts of alpha, beta, delta, and omega wolves to guide the search process and gradually approach the optimal solution [8]. The Harris Hawks Optimization algorithm (HHO) imitates the cooperative hunting behavior of Harris hawks. It combines various hunting strategies, such as surprise attacks, encirclement, and weakening the prey, to search for the global optimum in the solution space [9].

Currently, research in the field of swarm intelligence optimization algorithms mainly focuses on two directions. The first direction is the development of new algorithms with better performance. Researchers are constantly exploring new inspiration from nature and developing innovative algorithms that can handle complex optimization problems more effectively. These new algorithms often incorporate novel mechanisms and strategies to improve the search efficiency, convergence speed, and solution accuracy. The second direction is the improvement in the optimization efficiency of existing algorithms. This involves enhancing the update mechanisms of these algorithms, such as modifying the selection, crossover, and mutation operators, or introducing new strategies to balance exploration and exploitation. By improving the existing algorithms, researchers aim to make them more robust, efficient, and adaptable to different problem scenarios. These advancements in swarm intelligence optimization algorithms offer new ideas and tools for solving complex optimization problems in various fields and have the potential to revolutionize the way we approach optimization tasks [10,11,12].

The Enterprise Development Optimization (ED) algorithm is a unique intelligent optimization method inspired by the evolution mechanism of modern enterprises. In a dynamic market environment, enterprises strive for sustainable development through strategic adjustments, structural optimizations, technological upgrades, and personnel collaborations. The ED algorithm abstracts these real-world enterprise behaviors and decision-making processes into an optimization framework, aiming to find the best development paths for enterprises [13,14]. At the core of the ED algorithm is a dynamic equilibrium-based optimization framework. It simulates how enterprises balance market exploration (global search) and resource integration (local exploitation) under resource constraints. Market exploration helps enterprises identify new opportunities and expand their reach, while resource integration optimizes the allocation of internal resources to enhance operational efficiency. Additionally, the algorithm emphasizes maintaining organizational diversity through cross-departmental collaborations (population interactions) and strategic iterations (elite retention), mirroring the collaborative and adaptive nature of real-world enterprises [15,16]. However, like many other optimization algorithms, the ED algorithm has its limitations. Its solution accuracy can be relatively low when dealing with complex problems, often resulting in suboptimal solutions. Moreover, its convergence speed is often insufficient, which may hinder its practical application in time-sensitive scenarios. These drawbacks highlight the need for further research and improvement to enhance the algorithm’s performance and make it more applicable to real-world enterprise optimization tasks.

To solve the problems of low solution accuracy and slow convergence speed in the ED algorithm, this paper transforms the core behaviors of enterprises in response to the competitive environment into a mathematical model and proposes a Comprehensive Adaptive Enterprise Development Optimizer (CAED) that integrates an adaptive inertia weight strategy based on environmental sensitivity, a lens imaging reverse learning co-evolution strategy, and a chaos-perturbation-driven approach. By simulating the entire process of enterprises, dynamically adjusting their business structures, optimizing resource allocations, and responding to market fluctuations, the CAED enables efficient search in complex solution spaces and stable approximation to global optimal solutions. The algorithm shows significant advantages in decision-making problems with multi-dimensional constraints, such as organizational structure restructuring and supply chain optimization. The effectiveness and robustness of the CAED are verified by solving the optimal solutions of 23 typical test functions and the practical engineering cases of cantilever beam and three-bar truss designs.

This paper is organized as follows: Section 1 is the introduction, Section 2 is the research status of optimization algorithms, Section 3 introduces the enterprise optimization algorithm, Section 4 describes the improvement of the enterprise optimization algorithm, Section 5 presents the function performance test experiment and algorithm comparison, and Section 6 is the conclusion.

## 2. Research Status of Optimization Algorithms

Optimization algorithms tackle intricate engineering optimization problems by emulating the mechanisms present in natural ecosystems [17]. The relevant research can be broadly classified into three main categories. Firstly, there are general optimization algorithms. For example, the hill-climbing algorithm, which is grounded in the greedy strategy, steadily approaches the global optimum by way of local optimal solutions. Secondly, evolutionary algorithms exist, which mimic the mechanisms of biological evolution. Thirdly, swarm intelligence algorithms draw inspiration from the collaborative behaviors exhibited by group organisms [18,19]. The core concepts of these algorithms are essentially derived from the mathematical abstraction of natural laws, the process of biological evolution, and the concept of swarm intelligence [20]. Nevertheless, the first two types of algorithms tend to become stuck in local optimal solutions during the process of seeking solutions, and as a result, they are unable to attain the global optimum [21].

Swarm intelligence optimization algorithms address complex optimization problems by imitating the intelligent behaviors of biological groups. Their fundamental framework involves mapping optimization parameters to individual members of the group. Then, based on the collaborative rules of animals (such as foraging and migration behaviors), the states of these individuals are updated iteratively. The quality of the solutions is quantitatively evaluated through fitness functions. Ultimately, the global optimal solution is selected from the iterative process [22]. A distinctive characteristic of these algorithms is their utilization of group collaborative search strategies. This approach enables them to overcome the limitations of being trapped in local optima, making them particularly well suited for high-dimensional nonlinear optimization scenarios [23].

The ant colony optimization algorithm was initially introduced by the Italian scholar Marco Dorigo in his doctoral thesis in 1992. This algorithm is inspired by the way real ant colonies cooperate to find the shortest path by means of pheromones, and it serves as a typical example of swarm intelligence optimization algorithms [24]. In 1995, the American scholar James proposed the Particle Swarm Optimization algorithm. This algorithm models the collaborative behavior of bird flocks or fish schools when they are searching for food [25]. It considers the potential solutions of the optimization problem as “particles”, and these particles dynamically adjust their search directions by tracking the individual historical best solution (pbest) and the group historical best solution (gbest) in order to obtain the optimal solution. In 2003, the American scholar M. Eusuff put forward the shuffled frog-leaping algorithm. Inspired by the collaborative foraging behavior of frog groups in wetlands [26], the SFLA combines the concepts of memetic algorithms and Particle Swarm Optimization. It divides the population into multiple “sub-groups” (Memeplex), where each subgroup conducts a local search independently, and then global information exchange is achieved through population shuffling to attain the optimization effect [27]. In 2014, Chinese scholars Meng et al. proposed the chicken swarm optimization algorithm. Inspired by the hierarchical structure and foraging behavior within chicken flocks [28], the CSO regards the solutions of the optimization problem as “chicken flock individuals” and devises interaction rules based on the social hierarchy of chicken flocks (roosters, hens, chicks) to achieve the optimization goal [29].

In 2024, the enterprise optimization algorithm was proposed. It is an intelligent optimization algorithm that takes inspiration from the development process of modern enterprises. Its core inspiration stems from the strategies that enterprises adopt to achieve sustainable development. These strategies include task planning, structural adjustment, technological innovation, and human resource collaboration in a dynamic market environment [30,31]. The algorithm abstracts four crucial aspects of Enterprise Development, namely task, structure, technology, and people, into a mathematical model. It views the organizational structure as a workflow. The new organizational structure is influenced by the structures of other workflows within the organization as well as the current optimal workflow. This algorithm has been compared with several of the latest and renowned algorithms, and experimental results demonstrate that it can effectively solve complex multi-dimensional optimization problems.

## 3. Enterprise Optimization Algorithm

### 3.1. Personnel Initialization

Similarly to other common intelligent optimization algorithms, the enterprise optimization algorithm also starts with initialization. In the algorithm framework, each enterprise individual is regarded as a potential solution in the search space, and the entire enterprise *E* can be expressed by Equation (1) [32]:

(1) $$E=\left[\begin{array}{ccc} x_{1}^{1} & \cdots & x_{1}^{Dim}\\ \vdots & \ddots & \vdots \\ x_{pop}^{1} & \cdots & x_{pop}^{Dim} \end{array}\right]$$

In Equation (1), pop represents the total number of solutions, and each set of solutions consists of a Dim-dimensional vector group. The vector group and each personnel individual are defined as shown in Equation (2):

(2) $$\begin{array}{l} X_{i}=\left[x_{i}^{1},\cdots ,x_{i}^{Dim}\right]\\ x_{i}^{j}=rand\times \left(UB^{j}-LB^{j}\right)+LB^{j} \end{array}$$

In Equation (2), *Xi* is the *i*-th set of vector solutions, where *i* is in the range of [1,pop]; *X_ij_* is the *j*-th personnel individual in the *i*-th set of solutions, and *j* is in the range of [1,Dim]; *UB_j_* and *LBj* are the upper and lower limits of the *j*-th personnel individual, respectively; rand is a random number between [0,1].

The solution effect of intelligent optimization algorithms is usually measured by the fitness function *F*. Taking the problem of minimizing the fitness function as an example, the goal of this problem is to solve a set of *X_i_* such that the fitness function *F* reaches the minimum value.

At this time, *X_i_* is also defined as the optimal variable *X_best_*, as shown in Equation (3):

(3) $$X_{best}=argmin\left(F\left(X_{i}\right)\right)$$

In Equation (3), *F*(·) represents the fitness value of the problem to be solved.

### 3.2. Enterprise Business

#### 3.2.1. Optimal Rules for Enterprise Business Development

The Enterprise Development Optimization simulated by the algorithm needs to establish the following three core rules [33]: (1) A four-dimensional process dynamic switching mechanism. The ED process must include four core activities, task, structure, technology, and people, and establish a dynamic switching logic among the activities. (2) A performance-oriented strong-correlation design. All activities must be directly linked to organizational performance, and high-performance organizations can be preferentially strengthened in terms of the adaptability of the plan. (3) A quantitative evaluation system construction. The solutions must be systematically evaluated through a preset objective function to achieve an accurate measurement of organizational performance.

#### 3.2.2. Enterprise Business (Task)

Enterprise businesses come in different forms, such as daily affairs and external affairs. To simulate the task activities of an enterprise, the worst-case business is defined as shown in Equation (4):

(4) $$x_{worst}(t)=rand\times \left(UB-LB\right)+LB$$

In Equation (4), *x_worst_* represents the worst-case individual value in the solution space; *UB* and *LB* are the upper and lower limits of the solution space.

#### 3.2.3. Enterprise Structure (Structure)

In the enterprise optimization algorithm, the organizational structure of the enterprise is regarded as a workflow. The new organizational structure of the enterprise is affected by the structures of other workflows in the organization and the current optimal workflow. This process is updated by Equations (5) and (6):

(5) $$x_{i}^{s}(t)=x_{i}^{s}(t-1)+rand(-1,1)\times \left(x_{best}\left(t-1\right)-x_{c}^{s}\left(t-1\right)\right)$$

(6) $$x_{c}^{s}(t-1)=\frac{x_{rand1}^{s}\left(t-1\right)+x_{rand2}^{s}\left(t-1\right)+\cdots +x_{randm}^{s}\left(t-1\right)}{m}$$

In Equations (5) and (6), $x_{i}^{s}(t)$ represents the new organizational structure of the enterprise; $x_{best}\left(t-1\right)$ is the current optimal solution; $x_{c}^{s}(t-1)$ is the central organizational structure, which usually affects other new structures; $x_{randm}^{s}\left(t-1\right)$ is a randomly selected structure in the enterprise. Through experimental verification, when m is usually set to 3, the optimal result can be obtained in a relatively short calculation time.

#### 3.2.4. Enterprise Technology (Technology)

The technological level of an enterprise is usually considered the core factor affecting enterprise optimization. In most cases, the change in the enterprise’s organizational structure does not directly affect the enterprise’s optimization and development but rather promotes the enterprise’s technology to make breakthroughs in the new organizational structure. From the perspective of actual enterprise operations, the organizational structure of an enterprise balances the exploration and development efforts to obtain the necessary innovative technologies in the cruel market competition in the fastest and most effective way. Equation (7) simulates the process of balancing exploration and development:

(7) $$\begin{array}{l} x_{i}^{\tau }(t)=x_{i}^{\tau }(t-1)+rand_{\alpha }(0,1)\times \left(x_{best}\left(t-1\right)-x_{i}^{\tau }\left(t-1\right)\right)+\\ rand_{\beta }(0,1)\times \left(x_{best}\left(t-1\right)-x_{rand1}^{\tau }\left(t-1\right)\right) \end{array}$$

In Equation (7), $x_{i}^{\tau }(t)$ is the individual after a technology update; $x_{best}\left(t-1\right)-x_{i}^{\tau }\left(t-1\right)$ represents the adjustment of the algorithm’s exploration ability; $x_{best}\left(t-1\right)-x_{rand1}^{\tau }\left(t-1\right)$ represents the adjustment of the algorithm’s development ability.

#### 3.2.5. Enterprise People (People)

Enterprise personnel usually need to be trained before taking up their posts. Excellent enterprises can directly influence the value judgment and creative abilities of enterprise personnel through corporate culture. The corporate work culture also affects the work choices of enterprise personnel and determines the overall size of the enterprise. Assuming that the personnel characteristics are in one dimension, Equations (8) and (9) describe how to update the data characteristics of enterprise personnel by randomly selecting numbers to simulate the behavior of enterprise personnel:

(8) $$x_{i,d}^{p}(t)=x_{i,d}^{p}(t-1)+rand(-1,1)\times \left(x_{best,d}\left(t-1\right)-x_{c,d}^{p}\left(t-1\right)\right)$$

(9) $$x_{c,d}^{p}(t-1)=\frac{x_{rand1,d}^{p}\left(t-1\right)+x_{rand2}^{s}\left(t-1\right)+\cdots +x_{randm}^{s}\left(t-1\right)}{m}$$

In Equations (8) and (9), $x_{i,d}^{p}(t)$ is the advanced individual after personnel culture optimization; d is the random characteristic of the personnel, defined by Equation (10); m is the number of people affecting the personnel, and when m is set to 3, the optimization can be calculated in a relatively short amount of time.

(10) $$d=\left\lceil rand\left(0,1\right)\times n_{d}\right\rceil$$

In Equation (10), *n_d_* represents the dimension of the problem to be solved.

#### 3.2.6. Conversion Mechanism

Enterprise optimization usually does not carry out four steps simultaneously. Suppose an enterprise only focuses on one optimization step at a time, that is, only one task, structure, technology, and people change in the current iteration, and the iteration is controlled by the conversion mechanism. The conversion mechanism *c*(*t*) is defined as shown in Equation (11):

(11) $$c\left(t\right)=\left\lceil 3\times \left(1-\frac{rand\left(0,1\right)\times t}{Max_{iter}}\right)\right\rceil$$

In Equation (11), *t* represents the *t*-th task iteration; *Max_iter_* is the maximum number of iterations.

#### 3.2.7. Optimization Algorithm Process

**Figure 1:**
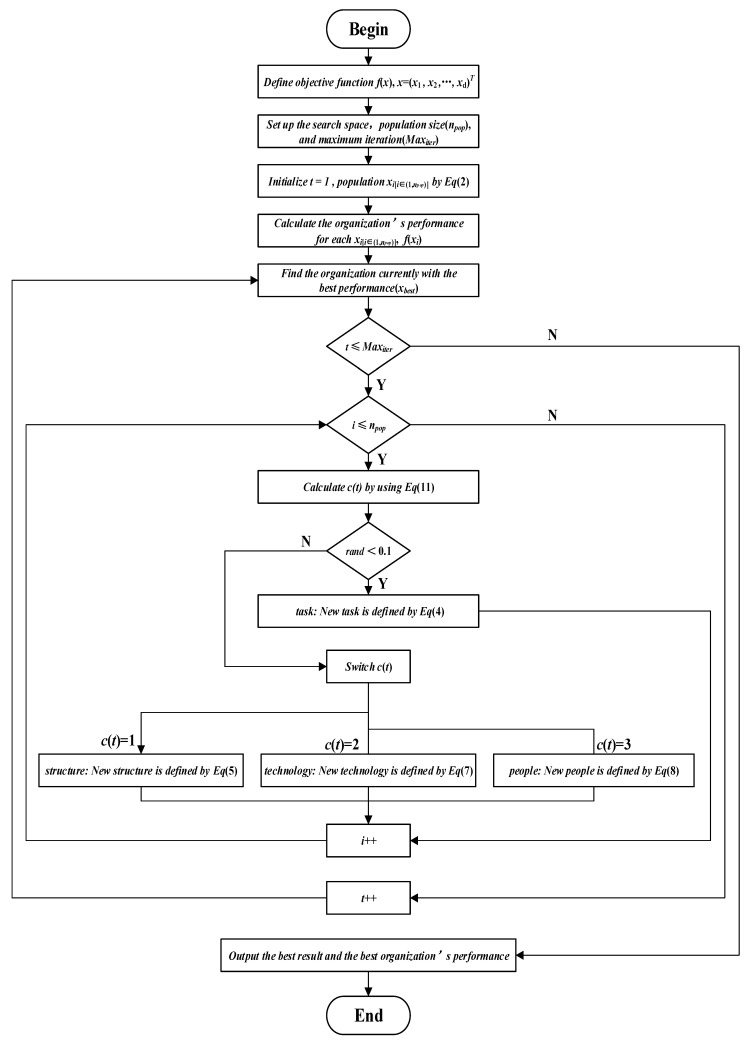
Enterprise optimization algorithm flow chart.

Combining data initialization and the four steps of enterprise optimization, the flow chart of the enterprise optimization algorithm is shown in Figure 1.

## 4. Comprehensive Adaptive Enterprise Optimization Algorithm

The enterprise optimization algorithm, a novel intelligent optimization approach, creates a distinctive population update mechanism by mimicking the adjustments in an enterprise’s development across business operations, organizational structure, technological aspects, and human resources. Nevertheless, this algorithm suffers from several issues, including the drawbacks of static step-size adjustment, the limitations of its one-way evolution mode, and inadequate responses to environmental changes. To address these problems and enhance its performance, this paper presents the Comprehensive Adaptive Enterprise Development Optimizer (CAED), which integrates multiple strategies. Firstly, the CAED utilizes the Tent chaotic map, which is based on random variables, for population initialization. By leveraging the mapping’s perturbation effect, it overcomes the periodic flaws of traditional sequences. This process generates uniformly distributed enterprise strategy solutions, effectively simulating the nonlinear nature of the market environment.

Secondly, the algorithm incorporates the lens imaging reverse learning strategy. Through performing interference jumps at the current implementation position, this strategy breaks free from the local-optimum constraints that typically limit traditional one-way adjustment methods. This enables the algorithm to explore a wider solution space and avoid becoming trapped in suboptimal solutions.

Moreover, the CAED constructs a dynamic search framework driven by an environmental sensitivity factor. This framework is integrated with an adaptive inertia weight strategy, allowing for the autonomous adjustment of the intensity between exploration and development. When the diversity of the population diminishes, the algorithm automatically boosts its global search capabilities, ensuring a more comprehensive exploration of the solution space and improving the overall optimization efficiency.

### 4.1. Tent Chaotic Map Based on Random Variables

The ED algorithm’s approach of randomly assigning the positions of its population inherently leads to an inevitable issue of uneven population distribution. When it comes to the search performance of the ED algorithm, there is a direct relationship, where a more uniform distribution of population individuals significantly increases the likelihood of discovering the optimal solution. Consequently, the initial configuration of the population becomes a pivotal factor in determining the algorithm’s effectiveness. Chaotic maps have gained substantial traction and widespread application in the domain of population initialization due to their unique properties [34,35]. Among them, the Tent chaotic map stands out with its remarkably straightforward mathematical representation and robust ergodicity. Its formula is presented in Equation (12), which showcases its simplicity and potential for efficient population initialization [36].

(12) $$Y_{i+1}=\left\{\begin{array}{c} \frac{Y_{i}}{a},\;\;\;0\leq Y_{i}<a\\ \frac{1-Y_{i}}{1-a},\;\;a\leq Y_{i}\leq 1 \end{array}\right.$$

**Figure 2:**
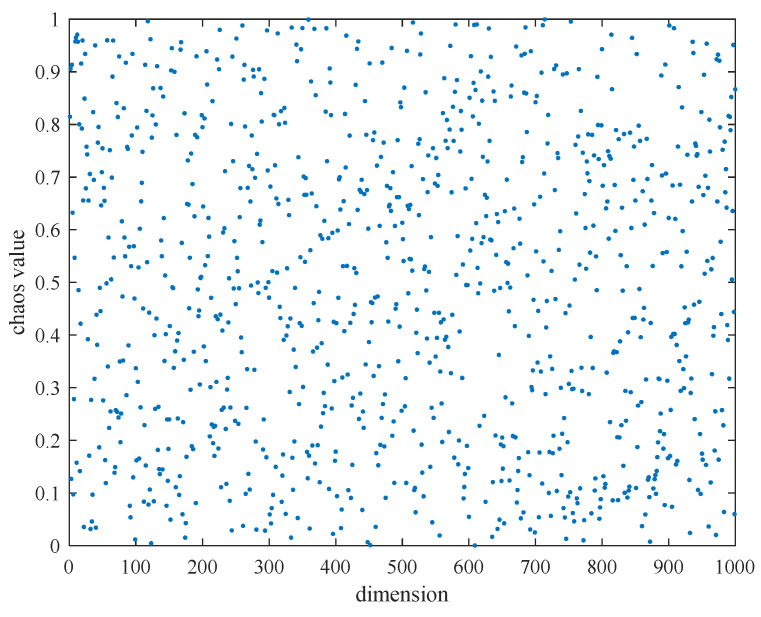
Tent chaos mapping sequence.

In Equation (12), *Y_i_* and *Y_i_*_+1_ are the chaotic values at the *i*-th and (*i* + 1)-th times, respectively. When *a* ∈ [0,1] and *Y_i_* ∈ [0,1], the system is in a chaotic state. When *a* is set to 0.3, the initial value *Y* is set to 0.32, and the number of iterations is set to 500 times, it can be seen that the Tent map is relatively evenly distributed in the [0,1] interval. The Tent chaotic map sequence in the [0,1] interval is shown in Figure 2.

The chaotic sequence generated by the Tent map has good distribution and randomness. By selecting multiple different initial values, a chaotic sequence of [0,1] is obtained to replace the random quantity in the population initialization of the ED algorithm, as shown in Equation (13), to complete the population initialization.

(13) $$X_{i}^{new}=(ub_{i}-lb_{i})Y_{i+1}+lb_{i}$$

In Equation (13), *ub_i_* and *lb_i_* are the upper and lower bounds of the independent variables of the objective function; $X_{i}^{new}$ is the position of enterprise personnel updated by the Tent chaotic map.

The pseudo-randomness and deterministic ergodicity (Lyapunov exponent > 0) of the Tent chaotic map enable the initial solutions to be distributed with low differences in the solution space. Compared with random initialization, the uniformity of the point set has been significantly improved, laying a high-quality solution-space foundation for the subsequent search stage of the algorithm.

### 4.2. Lens Imaging Reverse Learning Strategy

In the later stage of the ED algorithm operation, the species diversity gradually decreases, and individuals tend to gather around the optimal individual. If the optimal individual falls into a local optimal solution, it will be difficult for the population individuals to jump out of the local optimum, thereby reducing the optimization accuracy of the algorithm [37]. To address this challenge, this paper introduces a reverse learning strategy based on the lens imaging principle to perform interference operations on individuals, aiming to enhance the population diversity. This method aims to improve the algorithm’s ability to break free from the shackles of local optimal solutions and search for the global optimal solution more accurately [38,39].

This paper combines the converging lens imaging principle in the field of optics with the enterprise operation model. Suppose there is an individual in the interval [*lb*, *ub*], denoted as *P*, its height is set as *h*, and its projection on the *X*-axis is defined as *X* (this *X* is equivalent to the global optimal solution). The mid-point of [*lb*, *ub*] is marked as *O*, where a convex lens is placed, and its focal length is equal to *F*. When the individual *P* passes through the convex lens to produce a new image, an inverted image P* can be obtained at this time, and its size is h*. According to the standards of the convex lens imaging principle, the following conclusion can be drawn [40]:

(14) $$\frac{(ub+lb)/2-X}{X^{*}-(ub+lb)/2}=\eta$$

In Formula (14), $\eta =\frac{h}{h^{*}}$ is called the scaling factor. The inverse point can be obtained as follows:

(15) $$X^{*}=\frac{ub+lb}{2}+\frac{ub+lb}{2\eta }-\frac{X}{\eta }$$

After extending to the D-dimensional search space, the corresponding result can be obtained:

(16) $$X_{j}^{*}=ub_{j}+lb_{j}-X_{j}$$

In Formula (16), $X_{j}$ and $X_{j}^{*}$ are the *j*-th dimensional vectors of *X* and X*, respectively, and *ub_j_* and *lb_j_* are the *j*-th dimensional vectors of the decision variables. At the same time, this paper introduces a greedy mechanism to select the individuals after inverse learning so as to obtain the optimal individual. The mathematical model of the greedy mechanism is shown in Formula (17):

(17) $$X_{new}(t)=\left\{\begin{array}{c} X^{*},\;\;\;f(X)k\geq f(X^{*})\\ X,\;\;\;\;f(X)<f(X^{*}) \end{array}\right.$$

### 4.3. Adaptive Inertia Weight Strategy

The inertia weight *ω* reflects the ability of an individual to inherit the position of the previous individual. When the inertia weight value is large, it is helpful to enhance the exploration ability [41]. When the inertia weight is small, it is conducive to the specific exploitation ability. According to the principles of physics, the position of the *i*-th individual is updated based on the positions of the *i*-th and a random individual, which has a strong dependence on other individuals and is prone to becoming trapped in local optima and stagnating. In order to better balance the exploration and exploitation capabilities of the enterprise optimization algorithm, a linearly decreasing inertia weight is introduced, which determines the influence of previous individuals on the current individual. The new position update formula for the technology stage is as follows [42]:

(18) $$\begin{array}{l} x_{i}^{\tau }(t)=x_{i}^{\tau }(t-1)+rand^{\alpha }(0,1)\times \left(x_{best}(t-1)-x_{i}^{\tau }(t-1)\right)+\\ ws\times rand^{\beta }(0,1)\times \left(x_{best}(t-1)-x_{rand_{1}}^{\tau }(t-1)\right) \end{array}$$

(19) ω=(ωs−ωe)(T−t)/T+ωe

In Formulas (18) and (19), ωs is the initial inertia weight, ωe is the inertia weight when iterating to the maximum number of iterations, *t* is the current iteration number, and *T* is the maximum number of iterations.

Through experimental verification, it was determined that the algorithm has the best performance when ωs = 1 and ωe = 0.4. As the iteration progresses, the inertia weight linearly decreases from 1 to 0.4. A larger inertia weight at the beginning of the iteration enables the algorithm to maintain a good exploration ability, while a smaller inertia weight in the later stage of the iteration helps the algorithm to have a better exploitation ability.

### 4.4. Implementation Steps of the CAED

The Comprehensive Adaptive Enterprise Development Optimizer (CAED) introduces the Tent chaotic map to improve the distribution position of initialized individuals, the adaptive inertia weight strategy to optimize the individual update process, and the lens imaging inverse learning strategy to escape local optimal solutions in the later stage of the algorithm so as to find the global optimal solution more accurately. The implementation process and pseudo-code framework of the improved algorithm are as follows.

Step 1: Initialize relevant optimization parameters, set the dimension *D* of enterprise decision variables, the population size *N*, and the maximum number of iterations *Max_iter_*. Generate a Tent chaotic sequence and map the chaotic sequence to the solution space.

Step 2: Perform Tent chaotic map population initialization, calculate the inertia weight and transformation mechanism *c*(*t*) of the current iteration, and integrate the weight coefficient during the iteration strategy update.

Step 3: Refer to the content in Section 2 to execute the enterprise optimization algorithm process, update and recombine strategies, simulate the business restructuring of enterprise departments, and integrate resources according to conditions. Trigger the adjustment of structure, technology, and personnel under corresponding conditions, and retain the top 10% elite strategies for the next iteration.

Step 4: Lens imaging inverse learning (triggered in the later stage of iteration): When *t* reaches 0.7 Maxiter, perform inverse learning on the elite strategies, and select the individuals after inverse learning through the greedy mechanism to obtain the optimal individual.

Step 5: Output the optimal result when *Max_iter_* is reached.

**Figure 3:**
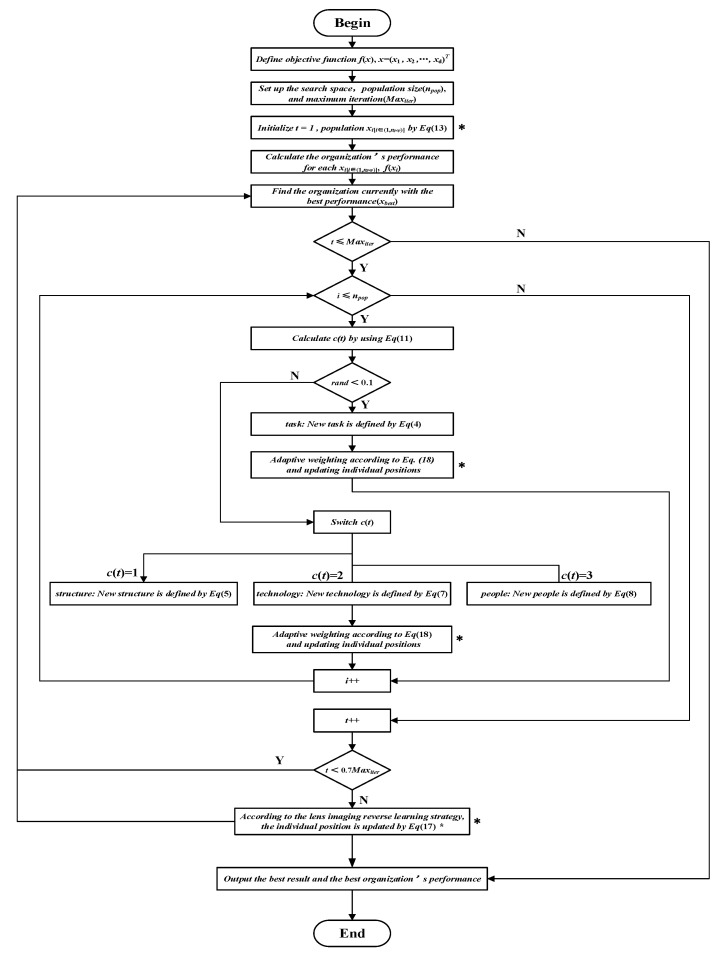
Flow chart of the CAED (* represents the improved steps).

The Flow chart of the CAED algorithm is shown in Figure 3. The pseudocode of comprehensive adaptive enterprise development optimizer is shown in Algorithm 1.
**Algorithm 1: Comprehensive Adaptive Enterprise Development Optimizer****Step 1: Initialization** *objective function f*(*x*), *x* = (*x*_1_, *x*_2_, …, *x*_d_)*^T^*, *population size* (*n_pop_*), and *maximum iteration* (*Max_iter_*) *search space*, *up and lp limits for initialization* *Initialize time*: *t* = *1* *Initialize population x_i_*(*i* = 1, 2, …, *n_pop_*) *by using Equation* (13) * The fitness value based on the objective function(*organization’s performance*) *Find the organization currently with the best fitness value(xbest)* **Repeat****Step 2:***Calculate the c*(*t*) *and ω according by Equations* (11) *and* (19), **go to Step 3****Step 3:****If** *rand* < *0.1*
* New task is defined by Equation* (4)
* Adaptive weighting according to Equation* (18) *and updating individual positions* *
**Else**
 **Switch** *c*(*t*)
    **Case** *c*(*t*) = 1
     *New structure is defined by Equation* (5)
     *Adaptive weighting according to Equation* (18) *and updating individual positions* *
    **Case** *c*(*t*) = 2 
     *New technology is defined by Equation* (7)
     *Adaptive weighting according to Equation* (18) *and updating individual positions* *
    **Case** *c*(*t*) = 3
     *New people is defined by Equation* (8)
     *Adaptive weighting according to Equation* (18) *and updating individual positions* *
 **End** *of switch*
**End** *of if*
*Update the organization currently with the best fitness value(xbest)*
*Update the time: t **++***
**If** *t* < 0.7*Max_iter_***, go to Step 3**
**Else If** *t* > 0.7*Max_iter_ and t* < *Max_iter_***: go to Step 4**
**Else: go to Step 5****Step 4***According to the lens imaging reverse learning strategy*, *the individual position is updated by Equation* (27) ***Step 5***Output the optimal solution*

## 5. Algorithm Performance Testing and Comparative Analysis

### 5.1. Experimental Design and Test Functions

To verify the search accuracy and robustness of the Comprehensive Adaptive Enterprise Development Optimizer (CAED) proposed in this paper when solving relevant optimization problems, the ED algorithm, CAED algorithm, PSO algorithm [43], GWO algorithm [44], AOA algorithm [45], DBO algorithm [46], GJO algorithm [47], SCSO algorithm [48], BKA algorithm [49], and SABO algorithm [50] were used to solve for the optimal values on 23 typical benchmark functions, and 50 independent experiments were carried out. The benchmark functions are shown in Table 1.

### 5.2. Experimental Results and Algorithm Analysis and Comparison

**Figure 4:**
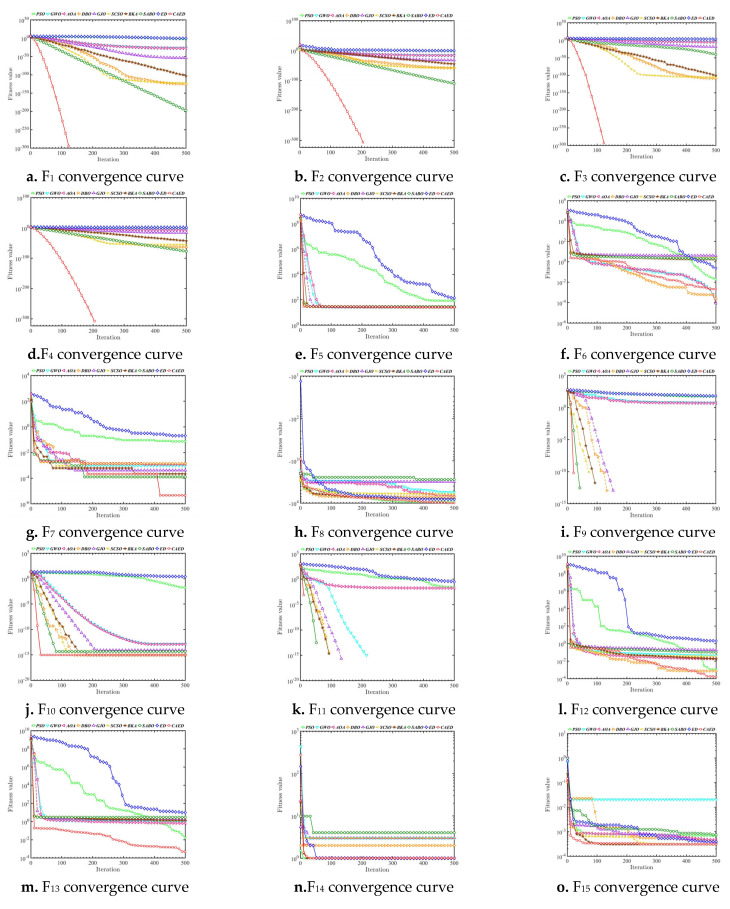

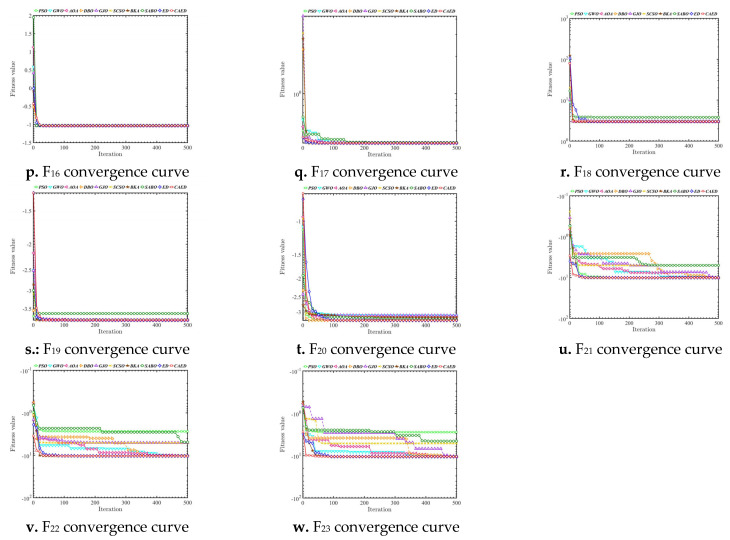
Benchmark function convergence curve.

**Figure 5:**
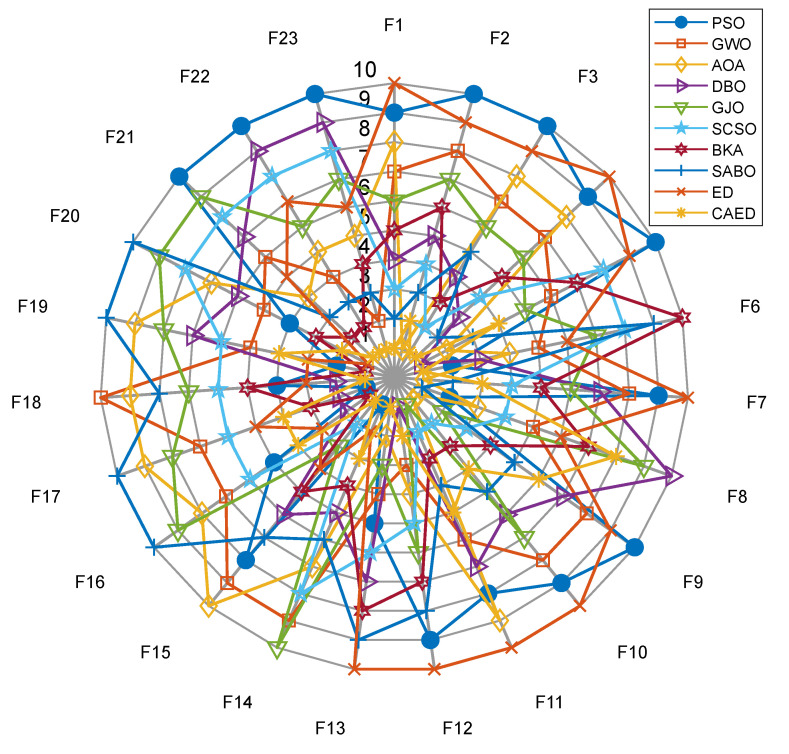
The radar charts of 10 algorithms in 23 test functions.

**Figure 6:**
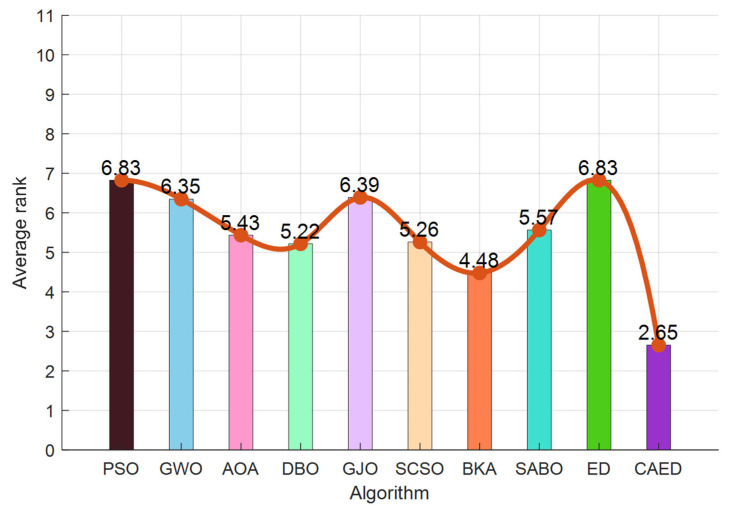
The average ranking questions of 10 algorithms in 23 test functions.

A comparison of the optimization results of benchmark functions F1–F23 is shown in Table 2 and Figure 4. A benchmark function convergence curve is shown in Figure 4. The radar charts and average ranking questions of 10 algorithms in 23 test functions are shown in Figure 5 and Figure 6.

Based on the test results of the 23 benchmark functions from CEC2005, the CAED demonstrates remarkable superiority in the vast majority of problems. For unimodal functions (such as F1–F5), the minimum values (min) of the CAED nearly all approach the theoretical optimal values (for example, the min of F1 is 0 and the min of F3 is 0), and the standard deviations (std) are extremely low (e.g., the std of F1 is 0). This indicates that the CAED can not only accurately approximate the global optimal solution but also exhibit high stability. Moreover, in multimodal functions (such as F8 and F21–F23), the worst values (worse) of the CAED are significantly better than those of other algorithms (for instance, the worse of F21 is −10.1532 for the CAED, while it is −2.6303 for GWO), suggesting that it can effectively avoid becoming trapped in local optima within complex search spaces. Although the computational time (time) of the CAED is slightly longer than that of some algorithms (e.g., the time of F1 is 0.0897 for the CAED and 0.0294 for PSO), its outstanding performance in optimization accuracy and robustness more than compensates for this drawback.

Other algorithms have their own advantages and disadvantages in different problems. DBO and BKA stand out in terms of stability. For example, the std of DBO in F1 is 3.73 × 10^−116^, and the std of BKA in F4 is 6.38 × 10^−77^. They are suitable for scenarios with high requirements for result consistency. GWO and PSO perform moderately in some high-dimensional problems (such as F8 and F20), but GWO has a relatively fast convergence speed (e.g., the time of F9 is 0.0583 for GWO), making it applicable to scenarios that demand quick responses. AOA and SABO perform poorly overall, especially in complex multimodal functions (for example, the mean value of SABO in F5 is 322.38, which is much higher than 26.62 of the CAED). This may be due to their sensitivity to parameters or insufficient search mechanisms, resulting in unstable performance. SCSO and GJO perform impressively in specific functions (e.g., the min of GJO in F7 is 5.12 × 10^−6^), but they have a high time cost (e.g., the time of SCSO in F6 is 0.6878). Thus, a trade-off between efficiency and accuracy is necessary.

Overall, the CAED, with its global search ability and stability, is the preferred algorithm for solving the CEC2005 benchmark problems, especially in scenarios that require high precision and robustness. If computational time is a concern, DBO or BKA can be considered as alternatives. Their stability is comparable to that of the CAED, and they consume less time (e.g., the time of BKA in F4 is 0.0685). For simple unimodal problems, GWO or PSO can also be used as supplementary options due to their high computational efficiency (e.g., the time of GWO in F10 is 0.0579). It is advisable to avoid using AOA and SABO, as they perform poorly in most problems and may affect the optimization results. Ultimately, the choice should be made by considering the complexity of the actual problem, accuracy requirements, and computational resource limitations.

### 5.3. Analysis of Algorithm Time Complexity

Assume that the population size of the enterprise optimization algorithm (ED) is *n*, the decision-making dimension is *j*, and the maximum number of iterations is *T_max_*. At the beginning, the algorithm randomly generates a population, so the time complexity of initializing the population is *O*(*n*·*j*). After entering the loop to find the optimal solution, in the strategy update stage, each of the j dimensions of each individual needs to be updated, and the time complexity of a single iteration is *O*(*n*·*j*). After updating the values of each individual, it is necessary to calculate the fitness value. Since the time complexity required for each individual is *O*(*j*), the total time complexity is *O*(*n*·*j*). After calculating the fitness values, it is necessary to find the optimal individual, and the time complexity at this time is *O*(*n*). In summary, the total time complexity of the enterprise optimization algorithm is shown in Equation (20):

(20) $$T_{ED}=O\left(n\cdot j\right)+T_{\max}\cdot O\left(n\cdot j\right)=O\left(T_{\max}\cdot n\cdot j\right)$$

In the Comprehensive Adaptive Enterprise Development Optimizer (CAED), the time complexity of generating a population using the Tent chaotic map is *O*(*n*·*j*). The time complexity of the adaptive inertia weight is *O*(1). In the lens imaging inverse learning, since the number of trigger times is 0.3*T_max_*, the time complexity of generating inverse solutions is *O*(0.3*n*·*j*). The time complexity of individual selection using the greedy mechanism is *O*(*nlogn*). After calculating the fitness values, the time complexity of finding the optimal individual is also *O*(*n*). In summary, after calculation and ignoring the lower-order terms, the total time complexity of the Comprehensive Adaptive Enterprise Development Optimizer (CAED) is shown in Equation (21):

(21) $$T_{CAED}=O\left(n\cdot j\right)+T_{\max}\left[O\left(n\cdot j\right)+0.3O\left(n\log n\right)\right]=O\left(T_{\max}\cdot n\cdot \left(j+\log n\right)\right)$$

Through the analysis of Equations (20) and (21), it can be seen that when solving high-dimensional problems, *j* >> *logn*. Therefore, in Equation (21), *j* + *logn* can be approximately regarded as *j*, and at this time, the time complexity is close to that of the original algorithm. From another perspective, even when using the CAED algorithm to solve high-dimensional problems, the time cost and the performance improvement after optimization are not of the same order of magnitude. That is, the CAED only incurs a slightly increased time cost (0.1% time cost), but it can bring about a significant performance boost.

### 5.4. Application of the CAED in Engineering

#### 5.4.1. Optimization of Cantilever Beam Design

Cantilever beam design is a typical optimization problem in structural engineering. Its aim is to determine the geometric parameters (such as cross-sectional shape and size) and material properties of the beam to achieve specific engineering goals (such as lightweight and low cost) while meeting mechanical performance requirements (strength, stiffness, and stability). A cantilever beam is usually fixed at one end and free at the other end, bearing external loads (such as concentrated forces and distributed forces), and it is necessary to avoid failures (such as yielding and excessive deformation). The goals and significance of optimizing the cantilever beam design are as follows:

1. Minimize the weight to reduce material usage and lower the self-weight of the structure. This is applicable in fields such as aerospace and automotive, saving material costs, improving energy efficiency, and enhancing the flexibility of movable structures.

2. Minimize the maximum stress/deformation to ensure that the stress of the beam does not exceed the allowable value of the material and the deformation is within the allowable range, guaranteeing structural safety and avoiding fatigue failure or functional failure.

3. Minimize the manufacturing cost by comprehensively considering material costs, processing complexity, and manufacturing process limitations. This can improve economic efficiency and meet the requirements of large-scale production.

The cantilever beam design problem is a structural engineering design problem related to the weight optimization of a square-cross-section cantilever beam. One end of the cantilever beam is rigidly supported, and a vertical force acts on the free node of the cantilever. The beam consists of five hollow square blocks with a constant thickness. Its height (or width) is the decision-making variable, and the thickness is fixed (here it is 2/3). This problem can be expressed by the following equations:

Objective function:

(22) $$f(X)=0.0624(x_{1}+x_{2}+x_{3}+x_{4}+x_{5})$$

Constraint conditions:

(23) $$g(X)=\frac{61}{x_{1}^{3}}+\frac{37}{x_{2}^{3}}+\frac{19}{x_{3}^{3}}+\frac{7}{x_{4}^{3}}+\frac{1}{x_{5}^{3}}-1\leq 0$$

Boundary constraints:

(24) $$0.01\leq x_{i}\leq 100,\;i=1,2,\cdots ,5$$

**Figure 7:**
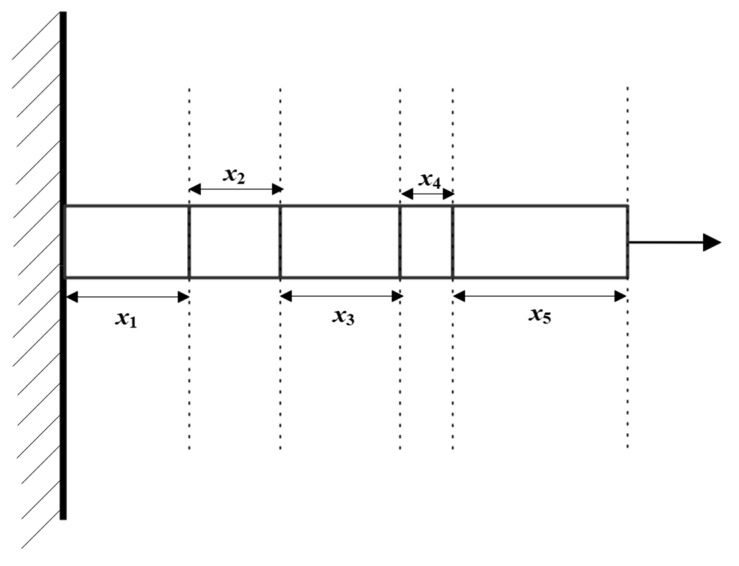
Schematic diagram of cantilever beam design structure.

The schematic diagram of cantilever beam design structure is shown in Figure 7.

**Figure 8:**
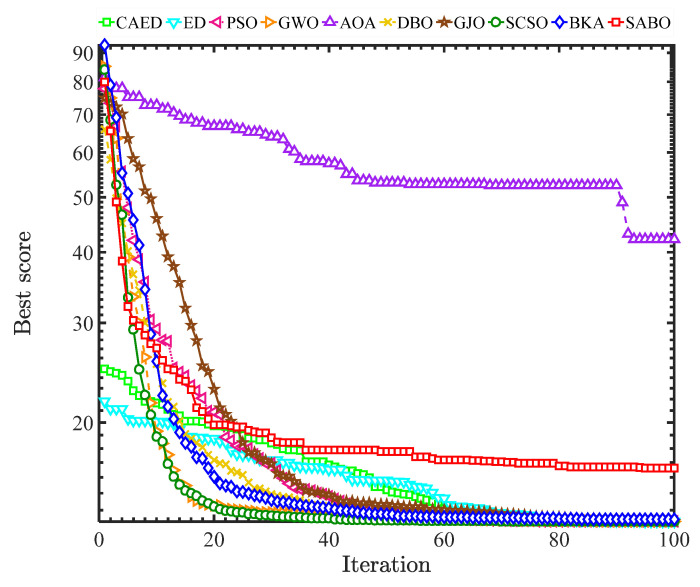
Cantilever beam convergence curve.

In the experimental simulation, 10 intelligent optimization algorithms were used, including the Comprehensive Adaptive Enterprise Development Optimizer (CAED), enterprise optimization algorithm (ED), Particle Swarm Optimization (PSO), Gray Wolf Optimizer (GWO), Arithmetic Optimization Algorithm (AOA), Dung Beetle Optimizer (DBO), Golden Jackal Optimizer (GJO), Sand Cat Swarm Optimizer (SCSO), Black-Winged Kite Optimizer (BKA), and Subtraction Average Optimizer (SABO). Simulation optimization experiments were carried out around the objective function of the cantilever beam design. The worst values, best values, standard deviations, average values, and median values of each optimization algorithm were recorded, as shown in Table 3, and the convergence curves are shown in Figure 8.

Based on the comprehensive analysis of the experimental results, in terms of the optimal solution (best), the CAED (13.3925) performs the best, outperforming the ED (13.4365), GWO (13.3661), etc., and is significantly better than the AOA (90.03) and SABO (18.8989). In terms of stability (std), the standard deviation of the CAED (0.0098) is the smallest, indicating the most stable results. The GWO (0.0013) and DBO (0.0077) follow. In terms of the average value (mean) and median value (median), the average value (13.3712) and median value (13.3696) of the CAED are both close to the optimal value, showing a balanced, comprehensive performance. In terms of the worst value (worst), the CAED (13.3605) performs the best among all algorithms. From the results, it can be seen that the CAED leads comprehensively in the cantilever beam design, especially in terms of stability and the quality of the optimal solution.

#### 5.4.2. Optimization of Three-Bar Truss Design

Three-bar truss design is a classic optimization problem in structural engineering. The goal is to determine the geometric parameters (such as the cross-sectional area, length, and node positions of the bars) and material properties of the truss structure composed of three bars to achieve goals, such as lightweight, low cost, or high reliability, while meeting mechanical performance constraints (such as strength, stiffness, and stability). Three-bar trusses are usually used in simple support structures (such as bridges and roof brackets), and it is necessary to avoid failure modes (such as bar yielding, buckling, and excessive node displacement). The goals and significance of optimizing the three-bar truss design are as follows:

1. Minimize the structural weight to reduce material consumption and lower the manufacturing cost. This is suitable for weight-sensitive scenarios (such as aerospace and mobile structures).

2. Minimize the maximum stress to ensure that the stress of all the bars does not exceed the allowable value of the material, preventing yielding or fracture.

3. Minimize the node displacement to control the displacement of the key nodes and avoid functional failure or excessive structural deformation.

The goal of the three-bar truss design problem is to minimize its volume while satisfying the stress constraints on each side of the truss members. This problem can be described by the cross-sectional area (X = [*x*_1_,*x*_2_] = [A1,A2]), and its mathematical model is as follows:

Objective function:

(25) $$f(X)=(2\sqrt{2}A_{1}+A_{2})\times l$$

Constraint conditions:

(26) $$g_{1}(X)=\frac{\sqrt{2}A_{1}+A_{2}}{\sqrt{2}A_{1}^{2}+2A_{1}A_{2}}P-\sigma \leq 0$$

(27) $$g_{2}(X)=\frac{A_{2}}{\sqrt{2}A_{1}^{2}+2A_{1}A_{2}}P-\sigma \leq 0$$

(28) $$g_{3}(X)=\frac{1}{A_{1}+\sqrt{2}A_{2}}P-\sigma \leq 0$$

Boundary constraints:

(29) $$0\leq A_{1},\;A_{2}\leq 1$$

where *l* = 100 cm; P = 2 kN/(cm^2^); *σ* = 2 kN/(cm^2^).

**Figure 9:**
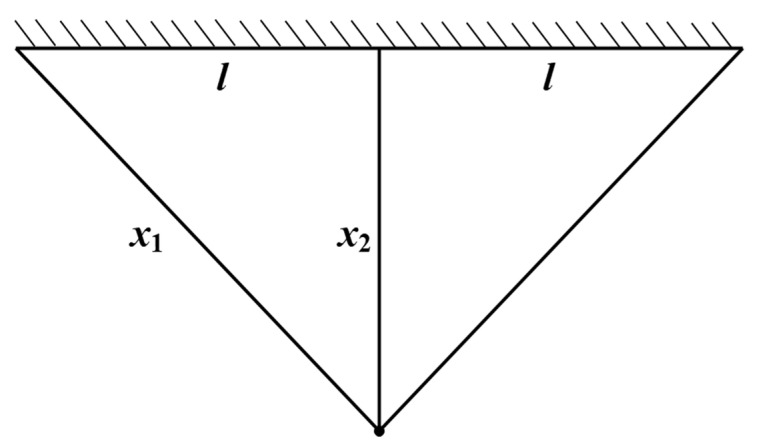
Schematic diagram of the three-bar truss design structure.

The schematic diagram of the three-bar truss design structure is shown in Figure 9.

**Figure 10:**
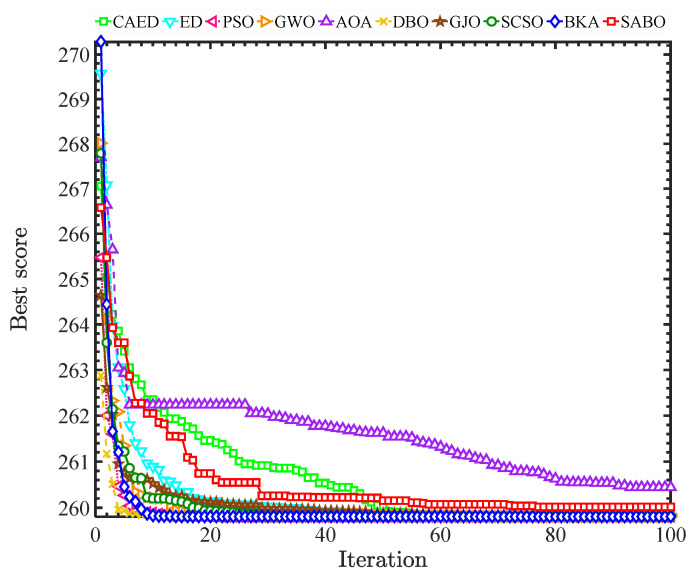
Cantilever beam convergence curve.

In the experimental simulation, 10 intelligent optimization algorithms were used, including the Comprehensive Adaptive Enterprise Development Optimizer (CAED), enterprise optimization algorithm (ED), Particle Swarm Optimization (PSO), Gray Wolf Optimizer (GWO), Arithmetic Optimization Algorithm (AOA), Dung Beetle Optimizer (DBO), Golden Jackal Optimizer (GJO), Sand Cat Swarm Optimizer (SCSO), Black-Winged Kite Optimizer (BKA), and Subtraction Average Optimizer (SABO). Simulation optimization experiments were carried out around the objective function of the three-bar truss design. The worst values, best values, standard deviations, average values, and median values of each optimization algorithm were recorded, as shown in Table 4, and the convergence curves are shown in Figure 10.

Based on the comprehensive analysis of the experimental results, in terms of the best solution (best), the CAED (259.805047) shows the best performance, while the AOA and SABO perform the worst (262.65 and 260.39, respectively), indicating that they are prone to becoming trapped in local optima. Regarding stability (std), the CAED has a relatively low standard deviation (1.11 × 10^−7^), outperforming most algorithms in terms of stability. In terms of the average value (mean) and median value (median), the mean and median values of the CAED, DBO, and BKA are the lowest (≈259.805), presenting the optimal comprehensive performance. Concerning the worst value (worst), the worst values of all the algorithms are close, but those of the AOA and SABO are significantly higher (259.85 and 259.82), suggesting that they perform poorly in the worst-case scenario. From these results, it can be seen that the CAED leads comprehensively in the three-bar truss design, excelling in optimization accuracy, stability, and convergence, and is thus the preferred algorithm for the three-bar truss design problem.

## 6. Conclusions

This paper puts forward the Comprehensive Adaptive Enterprise Development Optimizer (CAED), an innovative optimization algorithm designed to address the limitations of traditional enterprise optimization techniques. By integrating the Tent chaotic map initialization, lens imaging inverse learning, and a dynamic inertia weight strategy, the CAED effectively boosts the global search accuracy and convergence efficiency. The Tent chaotic map initialization helps in generating a diverse set of initial solutions, ensuring a more comprehensive exploration of the solution space from the start. The lens imaging inverse learning mechanism broadens the search scope by creating reverse solutions based on optical imaging principles, increasing the likelihood of escaping local optima. Meanwhile, the dynamic inertia weight strategy dynamically adjusts the balance between global exploration and local exploitation, enabling the algorithm to adaptively navigate through the different regions of the solution space.

A series of rigorous experiments were conducted to evaluate the performance of the CAED. The results clearly show that the CAED can closely approach the theoretical optimal solutions when applied to 23 benchmark functions. For instance, in the case of function F1, the standard deviation approaches zero, which strongly indicates its high precision and stability. In practical engineering applications, such as the design of a cantilever beam and a three-bar truss, the CAED significantly outperforms other comparative algorithms. For the cantilever beam problem, the CAED obtains an optimal value of 13.3925, while for the three-bar truss problem, it achieves an optimal value of 259.805047. These outstanding results firmly verify the robustness and practicality of the CAED in handling high-dimensional and complex problems, making it a promising solution for various real-world optimization tasks. However, like any algorithm, the CAED also has its limitations. In this paper, we thoroughly discuss the challenges that the algorithm may encounter when dealing with high-dimensional and complex problems. As the dimensionality of the problem increases, the computational complexity of the CAED rises significantly, which may lead to longer computation times and higher resource requirements. Additionally, the convergence speed of the algorithm may slow down, making it less efficient in finding the optimal solution within a reasonable time frame. We also analyze the possible failure scenarios of the algorithm in certain special situations. For example, when the objective function has a large number of local optimal solutions with a complex distribution, the CAED may struggle to distinguish the global optimal solution from the local ones, potentially resulting in suboptimal solutions.

In the section dedicated to scaling options, we explore various strategies to enhance the performance of the CAED algorithm. One approach is to further optimize and refine the existing strategies, such as improving the parameters and mechanisms of the Tent chaotic map initialization, lens imaging inverse learning, and dynamic inertia weight strategy. Another promising direction is to combine the CAED with other powerful optimization algorithms, leveraging their respective advantages to create a more efficient and effective hybrid algorithm. Looking ahead, future research on the CAED can focus on several key aspects. Multi-objective expansion would enable the algorithm to handle problems with multiple conflicting objectives simultaneously, which is common in many real-world applications. Improving its adaptability to dynamic scenarios would allow the CAED to respond effectively to changes in the problem environment in real time. Moreover, developing intelligent parameter tuning techniques would help the algorithm automatically adjust its parameters according to the characteristics of different problems, further enhancing its performance and versatility. These research directions have the potential to significantly expand the application depth of the CAED in important fields such as intelligent manufacturing and real-time scheduling, opening up new possibilities for solving complex optimization problems in these domains.

## Figures and Tables

**Table 1:** Benchmark functions table.

*Test Function*	*n*	*S*	*F_min_*
$F_{1}(x)=\sum_{i=1}^{n}x_{i}^{2}$	50	[−100,100]*^n^*	0
$F_{2}(x)=\sum_{i=1}^{n}\left|x_{i}\right|+\prod_{i=1}^{n}\left|x_{i}\right|$	50	[−10,10]*^n^*	0
$F_{3}(x)=\sum_{i=1}^{n}{\left(\sum_{j=1}^{i}x_{j}\right)}^{2}$	50	[−100,100]*^n^*	0
$F_{4}(x)=\max_{i}\left\{\left|x_{i}\right|,1\leq i\leq n\right\}$	50	[−100,100]*^n^*	0
$F_{5}(x)=\sum_{i=1}^{n-1}\left[100{\left(x_{i+1}-x_{i}^{2}\right)}^{2}+{\left(x_{i}-1\right)}^{2}\right]$	50	[−30,30]*^n^*	0
$F_{6}(x)=\sum_{i=1}^{n}{\left(\left\lfloor x_{i}+0.5\right\rfloor \right)}^{2}$	50	[−100,100]*^n^*	0
$F_{7}(x)=\sum_{i=1}^{n}ix_{i}^{4}+random\left[0,1\right)$	50	[−1.28,1.28]*^n^*	0
$F_{8}(x)=\sum_{i=1}^{n}-x_{i}\sin\left(\sqrt{\left|x_{i}\right|}\right)$	50	[−500,500]*^n^*	−12,569.5
$F_{9}(x)=\sum_{i=1}^{n}\left[x_{i}^{2}-10\cos\left(2\pi x_{i}\right)+10\right]$	50	[−5.12,5.12]*^n^*	0
$\begin{array}{l} F_{10}(x)=-20\exp\left(-0.2\sqrt{\frac{1}{n}}\sum_{i=1}^{n}x_{i}^{2}\right)-\exp\left(\frac{1}{n}\sum_{i=1}^{n}\cos2\pi x_{i}\right)\\ \;\;\;\;\;+\;\;\;20\;+\;e \end{array}$	50	[−32,32]*^n^*	0
$F_{11}(x)=\frac{1}{4000}\sum_{i=1}^{n}x_{i}^{2}-\prod_{i=1}^{n}\cos\left(\frac{x_{i}}{\sqrt{i}}\right)+1$	50	[−600,600]*^n^*	0
$\begin{array}{l} F_{12}(x)=\frac{\pi }{n}\left\{10\sin^{2}\left(\pi y_{i}\right)+\sum_{i=1}^{n-1}{\left(y_{i}-1\right)}^{2}\left[1+10\sin^{2}\left(\pi y_{i+1}\right)\right]\right.\\ \;\;\;\left.+{\left(y_{n}-1\right)}^{2}\right\}+\sum_{i=1}^{n}u\left(x_{i},10,100,4\right),\\ \;\;\;y_{i}=1+\frac{1}{4}\left(x_{i}+1\right)\\ \;\;\;u\left(x_{i},a,k,m\right)=\left\{\begin{array}{cc} k{\left(x_{i}-a\right)}^{m}, & x_{i}>a,\\ \begin{array}{c} 0,\\ k{\left(-x_{i}-a\right)}^{m}, \end{array} & \begin{array}{c} -a\leq x_{i}\leq a,\\ x_{i}<a. \end{array} \end{array}\right. \end{array}$	50	[−50,50]*^n^*	0
$\begin{array}{l} F_{13}(x)=0.1\left\{\sin^{2}\left(3\pi x_{1}\right)+\sum_{i=1}^{n-1}{\left(x_{i}-1\right)}^{2}\left[1+\sin^{2}\left(3\pi x_{i+1}\right)\right]\right.\\ \;\;\;\;\left.+{\left(x_{n}-1\right)}^{2}\left[1+\sin^{2}\left(2\pi x_{n}\right)\right]\right\}+\sum_{i=1}^{n}u\left(x_{1},5,100,4\right) \end{array}$	50	[−50,50]*^n^*	0
$F_{14}(x)={\left[\frac{1}{500}+\sum_{j=1}^{25}\frac{1}{j+\sum_{i=1}^{2}{\left(x_{i}-a_{ij}\right)}^{6}}\right]}^{-1}$	2	[−65.536, 65.536]*^n^*	0
$F_{15}(x)={\sum_{i=1}^{11}\left[a_{i}-\frac{x_{1}\left(b_{i}^{2}+b_{i}x_{2}\right)}{b_{i}^{2}+b_{i}x_{3}+x_{4}}\right]}^{2}$	4	[−5,5]*^n^*	0.000307
$F_{16}(x)=4x_{1}^{2}-2.1x_{1}^{4}+\frac{1}{3}x_{1}^{6}+x_{1}x_{2}-4x_{2}^{2}+4x_{2}^{4}$	2	[−5,5]*^n^*	−1.01362
$\begin{array}{l} F_{17}(x)={\left(x_{2}-\frac{5.1}{4\pi^{2}}x_{1}^{2}+\frac{5}{\pi }x_{1}-6\right)}^{2}+\\ \;\;\;\;\;10\left(1-\frac{1}{8\pi }\right)\cos x_{1}+10 \end{array}$	2	[−5,10] × [0,15]	0.398
$\begin{array}{l} F_{18}(x)=\left[1+{\left(x_{1}+x_{2}+1\right)}^{2}\left(19-14x_{1}+3x^{2}-14x_{2}\right.\right.\\ \;\;\;\;\;\left.\left.+6x_{1}x_{2}+3x_{2}^{2}\right)\right]\times \left[30+{\left(2x_{1}-3x_{2}\right)}^{2}\left(18-32x_{1}\right.\right.\\ \;\;\;\;\;\left.\left.+12x_{1}^{2}+48x_{2}-36x_{1}x_{2}+27x_{2}^{2}\right)\right] \end{array}$	2	[−2,2]*^n^*	3
$F_{19}(x)=-\sum_{i=1}^{4}c_{i}\exp\left[-\sum_{j=1}^{4}a_{ij}{\left(x_{j}-p_{ij}\right)}^{2}\right]$	4	[0,1]*^n^*	−3.86
$F_{20}(x)=-\sum_{i=1}^{4}c_{i}\exp\left[-\sum_{j=1}^{6}a_{ij}{\left(x_{j}-p_{ij}\right)}^{2}\right]$	6	[0,1]*^n^*	−3.32
$F_{21}(x)=-\sum_{i=1}^{5}{\left[\left(x-a_{i}\right){\left(x-a_{i}\right)}^{T}+c_{i}\right]}^{-1}$	4	[0,10]*^n^*	−10
$F_{22}(x)=-\sum_{i=1}^{7}{\left[\left(x-a_{i}\right){\left(x-a_{i}\right)}^{T}+c_{i}\right]}^{-1}$	4	[0,10]*^n^*	−10
$F_{23}(x)=-\sum_{i=1}^{10}{\left[\left(x-a_{i}\right){\left(x-a_{i}\right)}^{T}+c_{i}\right]}^{-1}$	4	[0,10]*^n^*	−10

**Table 2:** Comparison of optimization results of benchmark functions F1–F23.

F_1_	PSO	GWO	AOA	DBO	GJO	SCSO	BKA	SABO	ED	CAED
min	5.00 × 10^−4^	5.18 × 10^−29^	2.32 × 10^−158^	1.53 × 10^−194^	1.21 × 10^−57^	1.33 × 10^−124^	2.29 × 10^−107^	1.64 × 10^−201^	4.60 × 10^−3^	**0.00**
std	2.07 × 10^−2^	1.54 × 10^−27^	8.09 × 10^−38^	3.73 × 10^−116^	2.84 × 10^−54^	3.57 × 10^−108^	2.27 × 10^−77^	0.00	2.96 × 10^−1^	**0.00**
avg	8.89 × 10^−3^	1.22 × 10^−27^	1.48 × 10^−38^	6.82 × 10^−117^	1.96 × 10^−54^	6.52 × 10^−109^	4.15 × 10^−78^	8.01 × 10^−195^	2.56 × 10^−1^	**0.00**
median	3.72 × 10^−3^	5.66 × 10^−28^	3.48 × 10^−86^	7.51 × 10^−140^	7.10 × 10^−55^	2.36 × 10^−119^	3.04 × 10^−98^	1.07 × 10^−197^	1.07 × 10^−1^	**0.00**
worse	1.16 × 10^−1^	5.74 × 10^−27^	4.43 × 10^−37^	2.05 × 10^−115^	1.12 × 10^−53^	1.95 × 10^−107^	1.24 × 10^−76^	2.37 × 10^−193^	10.6	**0.00**
time	2.94 × 10^−2^	5.46 × 10^−2^	3.51 × 10^−2^	4.77 × 10^−2^	7.36 × 10^−2^	6.82 × 10^−1^	4.31 × 10^−2^	6.80 × 10^−2^	6.70 × 10^−2^	**8.97 × 10^−2^**
conv	1.00	1.00	1.00	1.00	1.00	1.00	1.00	1.00	0.00	**1.00**
**F_2_**	**PSO**	**GWO**	**AOA**	**DBO**	**GJO**	**SCSO**	**BKA**	**SABO**	**ED**	**CAED**
min	2.16 × 10^−3^	1.16 × 10^−17^	0	5.80 × 10^−82^	1.00 × 10^−33^	1.54 × 10^−65^	7.48 × 10^−54^	7.23 × 10^−113^	5.57 × 10^−2^	**0.00**
std	40.9	6.40 × 10^−17^	0	1.63 × 10^−53^	3.81 × 10^−32^	9.08 × 10^−60^	1.15 × 10^−44^	1.39 × 10^−110^	4.03 × 10^−1^	**0.00**
avg	20.3	8.16 × 10^−17^	0	2.98 × 10^−54^	2.20 × 10^−32^	1.87 × 10^−60^	2.09 × 10^−45^	6.74 × 10^−111^	3.10 × 10^−1^	**0.00**
median	2.04 × 10^−2^	6.16 × 10^−17^	0	6.59 × 10^−68^	8.68 × 10^−33^	9.08 × 10^−63^	5.97 × 10^−50^	1.41 × 10^−111^	1.97 × 10^−1^	**0.00**
worse	10.3	3.31 × 10^−16^	0	8.94 × 10^−53^	2.04 × 10^−31^	4.99 × 10^−59^	6.27 × 10^−44^	5.39 × 10^−110^	22.6	**0.00**
time	3.08 × 10^−2^	5.62 × 10^−2^	3.76 × 10^−2^	5.13 × 10^−2^	7.46 × 10^−2^	6.82 × 10^−1^	5.48 × 10^−2^	6.87 × 10^−2^	7.06 × 10^−2^	**9.13 × 10^−2^**
conv	1.00	1.00	1.00	1.00	1.00	1.00	1.00	1.00	0.00	**1.00**
**F_3_**	**PSO**	**GWO**	**AOA**	**DBO**	**GJO**	**SCSO**	**BKA**	**SABO**	**ED**	**CAED**
min	4.96 × 10^2^	9.60 × 10^−9^	8.20 × 10^−141^	7.93 × 10^−143^	3.67 × 10^−24^	6.02 × 10^−115^	4.20 × 10^−102^	1.23 × 10^−87^	1.53 × 10^3^	**0.00**
std	2.18 × 10^3^	3.80 × 10^−5^	7.86 × 10^−3^	5.50 × 10^−53^	3.44 × 10^−14^	5.37 × 10^−98^	2.09 × 10^−80^	7.80 × 10^−45^	1.24 × 10^3^	**0.00**
avg	2.51 × 10^3^	1.52 × 10^−5^	3.80 × 10^−3^	1.00 × 10^−53^	6.65 × 10^−15^	1.60 × 10^−98^	3.81 × 10^−81^	1.48 × 10^−45^	3.37 × 10^3^	**0.00**
median	1.60 × 10^3^	3.19 × 10^−6^	4.24 × 10^−36^	1.88 × 10^−116^	4.65 × 10^−20^	4.71 × 10^−103^	1.20 × 10^−96^	8.67 × 10^−62^	3.25 × 10^3^	**0.00**
worse	9.13 × 10^3^	1.97 × 10^−4^	2.57 × 10^−2^	3.01 × 10^−52^	1.89 × 10^−13^	2.23 × 10^−97^	1.14 × 10^−79^	4.28 × 10^−44^	6.37 × 10^3^	**0.00**
time	1.04 × 10^−1^	1.29 × 10^−1^	1.10 × 10^−1^	1.25 × 10^−1^	1.56 × 10^−1^	7.55 × 10^−1^	2.01 × 10^−1^	1.42 × 10^−1^	1.39 × 10^−1^	**2.34 × 10^−1^**
conv	0.00	1.00	1.00	1.00	1.00	1.00	1.00	1.00	0.00	**1.00**
**F_4_**	**PSO**	**GWO**	**AOA**	**DBO**	**GJO**	**SCSO**	**BKA**	**SABO**	**ED**	**CAED**
min	4.21 × 10	1.60 × 10^−7^	1.88 × 10^−66^	8.79 × 10^−84^	9.56 × 10^−19^	6.62 × 10^−56^	9.49 × 10^−53^	8.11 × 10^−79^	9.62 × 10	**0.00**
std	15.8	2.77 × 10^−6^	2.10 × 10^−2^	1.20 × 10^−53^	5.60 × 10^−15^	3.16 × 10^−48^	1.18 × 10^−42^	6.38 × 10^−77^	38.9	**0.00**
avg	68.4	1.57 × 10^−6^	2.35 × 10^−2^	2.19 × 10^−54^	1.72 × 10^−15^	5.85 × 10^−49^	2.18 × 10^−43^	3.75 × 10^−77^	22.6	**0.00**
median	64.8	5.28 × 10^−7^	3.70 × 10^−2^	1.26 × 10^−66^	6.97 × 10^−17^	1.27 × 10^−52^	7.73 × 10^−50^	1.43 × 10^−77^	22.6	**0.00**
worse	99.6	1.35 × 10^−5^	4.90 × 10^−2^	6.58 × 10^−53^	2.82 × 10^−14^	1.73 × 10^−47^	6.47 × 10^−42^	2.63 × 10^−76^	29.1	**0.00**
time	3.02 × 10^−2^	5.37 × 10^−2^	3.52 × 10^−2^	4.89 × 10^−2^	7.29 × 10^−2^	6.79 × 10^−1^	5.16 × 10^−2^	6.85 × 10^−2^	6.94 × 10^−2^	**8.77 × 10^−2^**
convergence	1.00	1.00	1.00	1.00	1.00	1.00	1.00	1.00	0.00	**1.00**
**F_5_**	**PSO**	**GWO**	**AOA**	**DBO**	**GJO**	**SCSO**	**BKA**	**SABO**	**ED**	**CAED**
min	29.4	25.2	27.0	25.2	26.5	26.0	26.0	27.9	93.9	**25.9**
std	1.64 × 10^4^	8.67 × 10^−1^	3.72 × 10^−1^	2.55 × 10^−1^	6.32 × 10^−1^	8.77 × 10^−1^	9.87 × 10^−1^	3.26 × 10^−1^	2.50 × 10^2^	**4.02 × 10^−1^**
avg	3.22 × 10^3^	26.9	28.4	25.8	27.8	27.8	27.7	28.5	3.22 × 10^2^	**26.6**
median	87.8	27.0	28.5	25.7	28.0	28.0	27.9	28.6	2.50 × 10^2^	**26.7**
worse	9.01 × 10^4^	28.7	28.9	26.5	28.8	28.8	28.9	28.9	1.43 × 10^3^	**27.3**
time	3.88 × 10^−2^	6.31 × 10^−2^	4.49 × 10^−2^	5.68 × 10^−2^	8.44 × 10^−2^	6.92 × 10^−1^	6.87 × 10^−2^	7.69 × 10^−2^	7.71 × 10^−2^	**1.03 × 10^−1^**
convergence	0.00	1.00	1.00	0.00	1.00	1.00	1.00	1.00	0.00	**0.00**
**F_6_**	**PSO**	**GWO**	**AOA**	**DBO**	**GJO**	**SCSO**	**BKA**	**SABO**	**ED**	**CAED**
min	3.71 × 10^−4^	2.58 × 10^−3^	2.62 × 10	3.26 × 10^−6^	17.5	4.98 × 10^−1^	9.75 × 10^−1^	14.1	4.54 × 10^−3^	**2.60 × 10^−4^**
std	1.15 × 10^−2^	3.44 × 10^−1^	2.40 × 10^−1^	4.48 × 10^−2^	4.73 × 10^−1^	5.88 × 10^−1^	1.39 × 10	6.37 × 10^−1^	2.25 × 10^−1^	**5.51 × 10^−3^**
avg	1.01 × 10^−2^	8.13 × 10^−1^	32.4	9.38 × 10^−3^	2.59 × 10	19.0	19.4	26.5	2.21 × 10^−1^	**4.29 × 10^−3^**
median	5.72 × 10^−3^	7.58 × 10^−1^	32.8	1.67 × 10^−4^	2.51 × 10	19.9	14.0	25.3	1.67 × 10^−1^	**2.23 × 10^−3^**
worse	5.61 × 10^−2^	1.27 × 10	36.9	2.46 × 10^−1^	3.73 × 10	27.9	67.7	39.3	7.66 × 10^−1^	**2.84 × 10^−2^**
time	3.00 × 10^−2^	5.34 × 10^−2^	3.56 × 10^−2^	4.75 × 10^−2^	7.32 × 10^−2^	6.88 × 10^−1^	5.06 × 10^−2^	6.82 × 10^−2^	6.99 × 10^−2^	**8.27 × 10^−2^**
convergence	1.00	1.00	1.00	1.00	1.00	1.00	1.00	1.00	1.00	**1.00**
**F_7_**	**PSO**	**GWO**	**AOA**	**DBO**	**GJO**	**SCSO**	**BKA**	**SABO**	**ED**	**CAED**
min	2.21 × 10^−2^	1.04 × 10^−3^	1.43 × 10^−6^	3.15 × 10^−5^	7.11 × 10^−5^	5.13 × 10^−6^	6.53 × 10^−6^	1.12 × 10^−5^	6.22 × 10^−2^	**2.01 × 10^−6^**
std	1.49 × 10^−2^	7.05 × 10^−4^	7.42 × 10^−5^	1.02 × 10^−3^	1.40 × 10^−3^	1.74 × 10^−4^	2.31 × 10^−4^	1.18 × 10^−4^	4.50 × 10^−2^	**1.54 × 10^−4^**
avg	5.05 × 10^−2^	2.05 × 10^−3^	8.34 × 10^−5^	1.14 × 10^−3^	6.64 × 10^−4^	1.26 × 10^−4^	2.60 × 10^−4^	1.52 × 10^−4^	1.25 × 10^−1^	**1.00 × 10^−4^**
median	5.14 × 10^−2^	1.89 × 10^−3^	7.73 × 10^−5^	1.02 × 10^−3^	3.81 × 10^−4^	9.24 × 10^−5^	1.86 × 10^−4^	1.19 × 10^−4^	1.12 × 10^−1^	**5.26 × 10^−5^**
worse	8.32 × 10^−2^	3.85 × 10^−3^	2.97 × 10^−4^	4.15 × 10^−3^	7.90 × 10^−3^	9.41 × 10^−4^	7.57 × 10^−4^	4.06 × 10^−4^	2.59 × 10^−1^	**8.11 × 10^−4^**
time	8.17 × 10^−2^	1.06 × 10^−1^	8.93 × 10^−2^	1.00 × 10^−1^	1.31 × 10^−1^	7.35 × 10^−1^	1.53 × 10^−1^	1.19 × 10^−1^	1.19 × 10^−1^	**1.85 × 10^−1^**
convergence	1.00	1.00	1.00	1.00	1.00	1.00	1.00	1.00	1.00	**1.00**
**F_8_**	**PSO**	**GWO**	**AOA**	**DBO**	**GJO**	**SCSO**	**BKA**	**SABO**	**ED**	**CAED**
min	−1.01 × 10^4^	−7.61 × 10^3^	−6.02 × 10^3^	−1.22 × 10^4^	−6.43 × 10^3^	−8.59 × 10^3^	−1.15 × 10^4^	−4.08 × 10^3^	−1.24 × 10^4^	**−1.18 × 10^4^**
std	6.40 × 10^2^	9.57 × 10^2^	4.15 × 10^2^	1.61 × 10^3^	1.16 × 10^3^	7.83 × 10^2^	1.54 × 10^3^	3.13 × 10^2^	1.32 × 10^3^	**8.87 × 10^2^**
avg	−8.31 × 10^3^	−5.95 × 10^3^	−5.30 × 10^3^	−8.51 × 10^3^	−4.26 × 10^3^	−6.85 × 10^3^	−8.45 × 10^3^	−3.06 × 10^3^	−1.03 × 10^4^	**−9.40 × 10^3^**
median	−8.34 × 10^3^	−6.05 × 10^3^	−5.32 × 10^3^	−8.40 × 10^3^	−4.06 × 10^3^	−6.84 × 10^3^	−8.31 × 10^3^	−3.03 × 10^3^	−1.06 × 10^4^	**−9.25 × 10^3^**
worse	−7.28 × 10^3^	−3.15 × 10^3^	−4.53 × 10^3^	−5.95 × 10^3^	−2.76 × 10^3^	−5.42 × 10^3^	−4.40 × 10^3^	−2.50 × 10^3^	−7.77 × 10^3^	**−7.80 × 10^3^**
time	4.06 × 10^−2^	6.64 × 10^−2^	4.89 × 10^−2^	6.28 × 10^−2^	8.63 × 10^−2^	6.95 × 10^−1^	7.57 × 10^−2^	7.94 × 10^−2^	8.29 × 10^−2^	**1.06 × 10^−1^**
convergence	1.00	0.00	1.00	0.00	0.00	0.00	0.00	1.00	0.00	**1.00**
**F_9_**	**PSO**	**GWO**	**AOA**	**DBO**	**GJO**	**SCSO**	**BKA**	**SABO**	**ED**	**CAED**
min	18.9	5.68 × 10^−14^	0.00	0.00	0.00	0.00	0.00	0.00	58.0	**0**
std	16.7	51.1	0.00	9.08 × 10^−1^	0.00	0.00	0.00	0.00	82.2	**0**
avg	51.0	37.4	0.00	1.66 × 10^−1^	0.00	0.00	0.00	0.00	72.1	**0**
median	47.8	15.6	0.00	0.00	0.00	0.00	0.00	0.00	72.7	**0**
worse	94.6	23.4	0.00	49.7	0.00	0.00	0.00	0.00	86.0	**0**
time	3.95 × 10^−2^	5.83 × 10^−2^	3.83 × 10^−2^	5.64 × 10^−2^	7.65 × 10^−2^	6.83 × 10^−1^	5.83 × 10^−2^	6.98 × 10^−2^	7.96 × 10^−2^	**9.06 × 10^−2^**
convergence	1.00	0.00	1.00	1.00	1.00	1.00	1.00	1.00	0.00	**1.00**
**F_10_**	**PSO**	**GWO**	**AOA**	**DBO**	**GJO**	**SCSO**	**BKA**	**SABO**	**ED**	**CAED**
min	9.29 × 10^−3^	7.55 × 10^−14^	8.88 × 10^−16^	8.88 × 10^−16^	4.44 × 10^−15^	8.88 × 10^−16^	8.88 × 10^−16^	4.44 × 10^−15^	1.80 × 10	**8.88 × 10^−16^**
std	7.31 × 10^−1^	1.64 × 10^−14^	0.00	9.01 × 10^−16^	1.53 × 10^−15^	0.00	0.00	0.00	9.97 × 10^−1^	**0.00**
avg	8.62 × 10^−1^	1.03 × 10^−13^	8.88 × 10^−16^	1.13 × 10^−15^	7.16 × 10^−15^	8.88 × 10^−16^	8.88 × 10^−16^	4.44 × 10^−15^	35.2	**8.88 × 10^−16^**
median	11.6	1.00 × 10^−13^	8.88 × 10^−16^	8.88 × 10^−16^	7.99 × 10^−15^	8.88 × 10^−16^	8.88 × 10^−16^	4.44 × 10^−15^	33.3	**8.88 × 10^−16^**
worse	21.3	1.36 × 10^−13^	8.88 × 10^−16^	4.44 × 10^−15^	7.99 × 10^−15^	8.88 × 10^−16^	8.88 × 10^−16^	4.44 × 10^−15^	57.3	**8.88 × 10^−16^**
time	3.88 × 10^−2^	5.79 × 10^−2^	3.97 × 10^−2^	5.35 × 10^−2^	7.69 × 10^−2^	6.85 × 10^−1^	5.74 × 10^−2^	7.06 × 10^−2^	7.97 × 10^−2^	**9.54 × 10^−2^**
convergence	1.00	1.00	1.00	1.00	1.00	1.00	1.00	1.00	0.00	**1.00**
**F_11_**	**PSO**	**GWO**	**AOA**	**DBO**	**GJO**	**SCSO**	**BKA**	**SABO**	**ED**	**CAED**
min	8.32 × 10^−4^	0.00	2.29 × 10^−2^	0.00	0.00	0.00	0.00	0.00	5.41 × 10^−2^	**0.00**
std	2.68 × 10^−2^	1.59 × 10^−2^	1.43 × 10^−1^	0.00	0.00	0.00	0.00	0.00	1.31 × 10^−1^	**0.00**
avg	3.15 × 10^−2^	5.66 × 10^−3^	1.98 × 10^−1^	0.00	0.00	0.00	0.00	0.00	2.37 × 10^−1^	**0.00**
median	2.37 × 10^−2^	0.00	1.74 × 10^−1^	0.00	0.00	0.00	0.00	0.00	2.17 × 10^−1^	**0.00**
worse	1.11 × 10^−1^	7.61 × 10^−2^	6.26 × 10^−1^	0.00	0.00	0.00	0.00	0.00	6.38 × 10^−1^	**0.00**
time	4.51 × 10^−2^	6.44 × 10^−2^	4.72 × 10^−2^	5.99 × 10^−2^	8.48 × 10^−2^	6.88 × 10^−1^	7.33 × 10^−2^	7.68 × 10^−2^	8.47 × 10^−2^	**1.07 × 10^−1^**
convergence	1.00	1.00	1.00	1.00	1.00	1.00	1.00	1.00	1.00	**1.00**
**F_12_**	**PSO**	**GWO**	**AOA**	**DBO**	**GJO**	**SCSO**	**BKA**	**SABO**	**ED**	**CAED**
min	1.07 × 10^−5^	1.33 × 10^−2^	4.20 × 10^−1^	8.57 × 10^−8^	6.91 × 10^−2^	3.70 × 10^−2^	2.55 × 10^−2^	8.75 × 10^−2^	1.49 × 10^−1^	**2.49 × 10^−5^**
std	1.85 × 10^−1^	2.44 × 10^−2^	4.87 × 10^−2^	1.62 × 10^−3^	1.24 × 10^−1^	4.30 × 10^−2^	1.89 × 10^−1^	9.00 × 10^−2^	17.6	**2.50 × 10^−4^**
avg	1.53 × 10^−1^	4.75 × 10^−2^	5.25 × 10^−1^	4.31 × 10^−4^	2.29 × 10^−1^	9.36 × 10^−2^	1.15 × 10^−1^	2.32 × 10^−1^	25.6	**2.15 × 10^−4^**
median	1.04 × 10^−1^	4.05 × 10^−2^	5.35 × 10^−1^	3.95 × 10^−6^	2.11 × 10^−1^	9.14 × 10^−2^	4.44 × 10^−2^	2.27 × 10^−1^	19.5	**1.12 × 10^−4^**
worse	7.28 × 10^−1^	1.01 × 10^−1^	6.11 × 10^−1^	6.86 × 10^−3^	7.56 × 10^−1^	2.24 × 10^−1^	7.27 × 10^−1^	4.05 × 10^−1^	58.4	**1.09 × 10^−3^**
time	1.63 × 10^−1^	1.87 × 10^−1^	1.70 × 10^−1^	1.85 × 10^−1^	2.37 × 10^−1^	8.16 × 10^−1^	3.20 × 10^−1^	2.00 × 10^−1^	1.94 × 10^−1^	**3.39 × 10^−1^**
convergence	1.00	1.00	1.00	1.00	1.00	1.00	1.00	1.00	1.00	**1.00**
**F_13_**	**PSO**	**GWO**	**AOA**	**DBO**	**GJO**	**SCSO**	**BKA**	**SABO**	**ED**	**CAED**
min	3.30 × 10^−4^	1.02 × 10^−1^	25.7	1.06 × 10^−5^	12.3	16.2	5.38 × 10^−1^	14.4	48.3	**4.32 × 10^−5^**
std	1.61 × 10^−1^	3.35 × 10^−1^	9.29 × 10^−2^	3.97 × 10^−1^	1.90 × 10^−1^	3.12 × 10^−1^	4.84 × 10^−1^	6.18 × 10^−1^	13.4	**3.41 × 10^−4^**
avg	1.25 × 10^−1^	6.23 × 10^−1^	28.3	4.31 × 10^−1^	16.7	24.3	17.3	24.1	22.6	**3.39 × 10^−4^**
median	6.61 × 10^−2^	6.31 × 10^−1^	28.4	3.71 × 10^−1^	16.7	25.0	17.3	28.5	20.2	**2.32 × 10^−4^**
worse	6.95 × 10^−1^	13.1	30.0	16.4	20.9	28.0	29.9	30.5	63.2	**1.45 × 10^−3^**
time	1.66 × 10^−1^	1.87 × 10^−1^	1.65 × 10^−1^	1.87 × 10^−1^	2.34 × 10^−1^	8.10 × 10^−1^	3.19 × 10^−1^	2.01 × 10^−1^	1.94 × 10^−1^	**3.36 × 10^−1^**
convergence	1.00	1.00	1.00	1.00	1.00	1.00	1.00	1.00	1.00	**1.00**
**F_14_**	**PSO**	**GWO**	**AOA**	**DBO**	**GJO**	**SCSO**	**BKA**	**SABO**	**ED**	**CAED**
min	9.98 × 10^−1^	9.98 × 10^−1^	1.99 × 10	9.98 × 10^−1^	9.98 × 10^−1^	9.98 × 10^−1^	9.98 × 10^−1^	10.0	9.98 × 10^−1^	**9.98 × 10^−1^**
std	5.83 × 10^−17^	32.2	36.5	9.23 × 10^−1^	41.8	30.9	5.03 × 10^−1^	15.4	0.00	**8.25 × 10^−17^**
avg	9.98 × 10^−1^	30.6	10.1	13.9	40.7	33.6	11.3	29.4	9.98 × 10^−1^	**9.98 × 10^−1^**
median	9.98 × 10^−1^	24.9	12.7	9.98 × 10^−1^	29.8	29.8	9.98 × 10^−1^	29.8	9.98 × 10^−1^	**9.98 × 10^−1^**
worse	9.98 × 10^−1^	12.7	12.7	49.5	12.7	10.8	29.8	61.8	9.98 × 10^−1^	**9.98 × 10^−1^**
time	2.48 × 10^−1^	2.45 × 10^−1^	2.47 × 10^−1^	2.74 × 10^−1^	2.73 × 10^−1^	2.92 × 10^−1^	5.02 × 10^−1^	2.66 × 10^−1^	2.88 × 10^−1^	**5.24 × 10^−1^**
convergence	1.00	1.00	1.00	1.00	1.00	1.00	1.00	1.00	1.00	**1.00**
**F_15_**	**PSO**	**GWO**	**AOA**	**DBO**	**GJO**	**SCSO**	**BKA**	**SABO**	**ED**	**CAED**
min	3.11 × 10^−4^	3.09 × 10^−4^	3.50 × 10^−4^	3.07 × 10^−4^	3.08 × 10^−4^	3.07 × 10^−4^	3.07 × 10^−4^	3.18 × 10^−4^	4.40 × 10^−4^	**3.07 × 10^−4^**
std	7.42 × 10^−3^	8.54 × 10^−3^	2.90 × 10^−2^	4.20 × 10^−4^	6.07 × 10^−3^	3.19 × 10^−4^	5.06 × 10^−3^	2.10 × 10^−3^	2.39 × 10^−4^	**7.71 × 10^−9^**
avg	4.08 × 10^−3^	5.14 × 10^−3^	2.05 × 10^−2^	9.91 × 10^−4^	2.47 × 10^−3^	4.40 × 10^−4^	1.78 × 10^−3^	9.26 × 10^−4^	1.08 × 10^−3^	**3.07 × 10^−4^**
median	7.30 × 10^−4^	4.30 × 10^−4^	1.01 × 10^−2^	1.22 × 10^−3^	4.71 × 10^−4^	3.08 × 10^−4^	3.07 × 10^−4^	4.80 × 10^−4^	1.22 × 10^−3^	**3.07 × 10^−4^**
worse	2.04 × 10^−2^	2.04 × 10^−2^	1.01 × 10^−1^	1.66 × 10^−3^	2.04 × 10^−2^	1.60 × 10^−3^	2.04 × 10^−2^	1.20 × 10^−2^	1.23 × 10^−3^	**3.08 × 10^−4^**
time	1.73 × 10^−2^	2.08 × 10^−2^	2.01 × 10^−2^	4.61 × 10^−2^	4.22 × 10^−2^	1.05 × 10^−1^	4.29 × 10^−2^	3.84 × 10^−2^	6.69 × 10^−2^	**8.48 × 10^−2^**
convergence	1.00	1.00	1.00	1.00	1.00	1.00	1.00	1.00	1.00	**1.00**
**F_16_**	**PSO**	**GWO**	**AOA**	**DBO**	**GJO**	**SCSO**	**BKA**	**SABO**	**ED**	**CAED**
min	−10.3	−10.3	−10.3	−10.3	−10.3	−10.3	−10.3	−10.3	−10.3	**−10.3**
std	6.52 × 10^−16^	1.59 × 10^−8^	1.42 × 10^−7^	5.68 × 10^−16^	1.87 × 10^−7^	1.15 × 10^−9^	5.76 × 10^−16^	1.40 × 10^−2^	6.52 × 10^−16^	**6.32 × 10^−16^**
avg	−10.3	−10.3	−10.3	−10.3	−10.3	−10.3	−10.3	−10.2	−10.3	**−10.3**
median	−10.3	−10.3	−10.3	−10.3	−10.3	−10.3	−10.3	−10.3	−10.3	**−10.3**
worse	−10.3	−10.3	−10.3	−10.3	−10.3	−10.3	−10.3	−9.86^−1^	−10.3	**−10.3**
time	1.75 × 10^−2^	1.88 × 10^−2^	1.85 × 10^−2^	4.35 × 10^−2^	3.81 × 10^−2^	6.12 × 10^−2^	3.83 × 10^−2^	3.64 × 10^−2^	6.54 × 10^−2^	**8.52 × 10^−2^**
convergence	1.00	1.00	1.00	1.00	1.00	1.00	1.00	1.00	1.00	**1.00**
**F_17_**	**PSO**	**GWO**	**AOA**	**DBO**	**GJO**	**SCSO**	**BKA**	**SABO**	**ED**	**CAED**
min	3.98 × 10^−1^	3.98 × 10^−1^	3.99 × 10^−1^	3.98 × 10^−1^	3.98 × 10^−1^	3.98 × 10^−1^	3.98 × 10^−1^	3.98 × 10^−1^	3.98 × 10^−1^	**3.98 × 10^−1^**
std	0.00	3.47 × 10^−4^	9.23 × 10^−3^	0.00	2.00 × 10^−3^	3.86 × 10^−8^	1.95 × 10^−15^	1.27 × 10^−1^	0.00	**0.00**
avg	3.98 × 10^−1^	3.98 × 10^−1^	4.09 × 10^−1^	3.98 × 10^−1^	3.98 × 10^−1^	3.98 × 10^−1^	3.98 × 10^−1^	4.48 × 10^−1^	3.98 × 10^−1^	**3.98 × 10^−1^**
median	3.98 × 10^−1^	3.98 × 10^−1^	4.06 × 10^−1^	3.98 × 10^−1^	3.98 × 10^−1^	3.98 × 10^−1^	3.98 × 10^−1^	4.01 × 10^−1^	3.98 × 10^−1^	**3.98 × 10^−1^**
worse	3.98 × 10^−1^	4.00 × 10^−1^	4.34 × 10^−1^	3.98 × 10^−1^	4.09 × 10^−1^	3.98 × 10^−1^	3.98 × 10^−1^	1.00	3.98 × 10^−1^	**3.98 × 10^−1^**
time	1.29 × 10^−2^	1.45 × 10^−2^	1.53 × 10^−2^	4.15 × 10^−2^	3.47 × 10^−2^	5.72 × 10^−2^	3.44 × 10^−2^	3.17 × 10^−2^	6.36 × 10^−2^	**7.69 × 10^−2^**
convergence	1.00	1.00	1.00	1.00	1.00	1.00	1.00	1.00	1.00	**1.00**
**F_18_**	**PSO**	**GWO**	**AOA**	**DBO**	**GJO**	**SCSO**	**BKA**	**SABO**	**ED**	**CAED**
min	3.00	3.00	3.00	3.00	3.00	3.00	3.00	3.00	3.00	**3.00**
std	14.8	41.0^−5^	11.0	49.3	1.99 × 10^−6^	1.38 × 10^−5^	2.31 × 10^−15^	16.0	1.06 × 10^−15^	**1.59 × 10^−15^**
avg	57.0	3.00	84.0	39.0	3.00	3.00	3.00	39.7	3.00	**3.00**
median	300	3.00	3.00	3.00	3.00	3.00	3.00	32.6	3.00	**3.00**
worse	84.0	3.00	30.0	30.0	3.00	3.00	3.00	87.7	3.00	**3.00**
time	1.25 × 10^−2^	1.39 × 10^−2^	1.36 × 10^−2^	3.90 × 10^−2^	3.37 × 10^−2^	5.62 × 10^−2^	3.37 × 10^−2^	3.15 × 10^−2^	6.33 × 10^−2^	**7.62 × 10^−2^**
convergence	1.00	1.00	1.00	1.00	1.00	1.00	1.00	1.00	1.00	**1.00**
**F_19_**	**PSO**	**GWO**	**AOA**	**DBO**	**GJO**	**SCSO**	**BKA**	**SABO**	**ED**	**CAED**
min	−38.6	−38.6	−38.6	−38.6	−38.6	−38.6	−38.6	−38.6	−38.6	**−38.6**
std	2.60 × 10^−15^	2.15 × 10^−3^	3.80 × 10^−3^	3.21 × 10^−3^	3.92 × 10^−3^	3.20 × 10^−3^	2.40 × 10^−15^	2.41 × 10^−1^	2.71 × 10^−15^	**2.71 × 10^−15^**
avg	−38.6	−38.6	−38.5	−38.6	−38.6	−38.6	−38.6	−35.6	−38.6	**−38.6**
median	−38.6	−38.6	−38.5	−38.6	−38.6	−38.6	−38.6	−36.1	−38.6	**−38.6**
worse	−38.6	−38.5	−38.4	−3.85	−38.5	−38.5	−38.6	−29.8	−38.6	**−38.6**
time	1.99 × 10^−2^	2.26 × 10^−2^	2.22 × 10^−2^	4.88 × 10^−2^	4.33 × 10^−2^	8.59 × 10^−2^	4.90 × 10^−2^	4.06 × 10^−2^	7.21 × 10^−2^	**9.41 × 10^−2^**
convergence	1.00	1.00	1.00	1.00	1.00	1.00	1.00	1.00	1.00	**1.00**
**F_20_**	**PSO**	**GWO**	**AOA**	**DBO**	**GJO**	**SCSO**	**BKA**	**SABO**	**ED**	**CAED**
min	−33.2	−33.2	−31.5	−33.2	−33.2	−33.2	−332	−33.2	−33.2	**−33.2**
std	6.54 × 10^−2^	1.00 × 10^−1^	8.73 × 10^−2^	1.05 × 10^−1^	9.09 × 10^−2^	1.18 × 10^−1^	6.03 × 10^−2^	1.60 × 10^−1^	1.36 × 10^−15^	**1.42 × 10^−15^**
avg	−32.8	−32.3	−30.4	−32.4	−31.7	−32.4	−32.9	−32.2	−33.2	**−33.2**
median	−33.2	−32.6	−30.5	−33.2	−31.3	−33.2	−33.2	−33.1	−33.2	**−33.2**
worse	−31.4	−30.2	−28.4	−28.5	−30.2	−28.4	−31.2	−25.9	−33.2	**−33.2**
time	2.30 × 10^−2^	2.72 × 10^−2^	2.46 × 10^−2^	5.01 × 10^−2^	5.01 × 10^−2^	1.53 × 10^−1^	5.11 × 10^−2^	4.43 × 10^−2^	7.41 × 10^−2^	**9.30 × 10^−2^**
convergence	1.00	1.00	1.00	1.00	1.00	1.00	1.00	1.00	1.00	**1.00**
**F_21_**	**PSO**	**GWO**	**AOA**	**DBO**	**GJO**	**SCSO**	**BKA**	**SABO**	**ED**	**CAED**
min	−10.2	−10.2	−56.1	−10.2	−10.2	−10.2	−10.2	−50.5	−10.1	**−10.2**
std	35.9	23.7	7.32 × 10^−1^	26.9	29.1	22.1	2.39 × 10^−6^	5.69 × 10^−1^	22.3	**5.96 × 10^−15^**
avg	−69.3	−88.9	−35.3	−75.4	−78.7	−53.8	−10.2	−47.9	−77.4	**−10.2**
median	−10.2	−10.2	−34.8	−76.2	−10.1	−50.6	−10.2	−50.5	−88.8	**−10.2**
worse	−26.3	−26.3	−22.4	−26.3	−26.3	−8.82 × 10^−1^	−10.2	−28.8	−50.6	**−10.2**
time	2.64 × 10^−2^	2.84 × 10^−2^	2.77 × 10^−2^	5.39 × 10^−2^	4.98 × 10^−2^	1.13 × 10^−1^	5.94 × 10^−2^	4.79 × 10^−2^	7.76 × 10^−2^	**1.01 × 10^−1^**
convergence	1.00	1.00	1.00	1.00	0.00	1.00	1.00	1.00	1.00	**1.00**
**F_22_**	**PSO**	**GWO**	**AOA**	**DBO**	**GJO**	**SCSO**	**BKA**	**SABO**	**ED**	**CAED**
min	−10.4	−10.4	−10.1	−10.4	−10.4	−10.4	−10.4	−50.9	−10.4	**−10.4**
std	297	1.08 × 10^−3^	192	268	134	265	6.17 × 10^−5^	4.66 × 10^−1^	2.39	**9.33 × 10^−16^**
avg	−86.8	−10.4	−44.4	−85.6	−10.0	−69.9	−10.4	−48.0	−79.0	**−10.4**
median	−10.4	−10.4	−42.7	−10.4	−10.4	−50.9	−10.4	−50.5	−93.6	**−10.4**
worse	−27.7	−10.4	−12.5	−27.7	−50.9	−37.2	−10.4	−31.9	−50.9	**−10.4**
time	3.06 × 10^−2^	3.30 × 10^−2^	3.22 × 10^−2^	5.77 × 10^−2^	5.42 × 10^−2^	1.17 × 10^−1^	6.78 × 10^−2^	5.03 × 10^−2^	8.02 × 10^−2^	**1.11 × 10^−1^**
convergence	1.00	0.00	1.00	1.00	0.00	1.00	1.00	1.00	1.00	**1.00**
**F_23_**	**PSO**	**GWO**	**AOA**	**DBO**	**GJO**	**SCSO**	**BKA**	**SABO**	**ED**	**CAED**
min	−10.5	−10.5	−63.7	−10.5	−10.5	−10.5	−10.5	−97.5	−10.5	**−10.5**
std	356	9.79 × 10^−1^	12.3	27.4	21.8	26.1	16.4	11.0	22.3	**1.14^−15^**
avg	−82.6	−10.4	−37.4	−89.4	−98.2	−66.7	−10.1	−48.5	−90.0	**−10.5**
median	−10.5	−10.5	−37.9	−10.5	−10.5	−51.3	−10.5	−48.7	−10.2	**−10.5**
worse	−24.2	−51.7	−17.6	−28.1	−24.2	−28.1	−33.3	−28.0	−51.3	**−10.5**
time	3.65 × 10^−2^	3.94 × 10^−2^	3.87 × 10^−2^	6.44 × 10^−2^	6.10 × 10^−2^	1.24 × 10^−1^	7.64 × 10^−2^	5.72 × 10^−2^	8.81 × 10^−2^	**1.23 × 10^−1^**
convergence	1.00	0.00	1.00	1.00	0.00	1.00	1.00	1.00	1.00	**1.00**

**Table 3:** Comparison table of optimization results for cantilever beam design.

Cantilever Beam	CAED	ED	PSO	GWO	AOA	DBO	GJO	SCSO	BKA	SABO
worst	13.3605	13.3621	13.3616	13.3619	22.8580	13.3622	13.3695	13.3604	13.3606	14.7054
best	13.3925	13.4365	13.3737	13.3661	90.0301	13.3892	13.4033	13.3812	14.7070	18.8989
std	0.0098	0.0248	0.0045	0.0013	19.7027	0.0077	0.0119	0.0065	0.4254	1.4752
mean	13.3712	13.3830	13.3670	13.3636	42.1413	13.3707	13.3817	13.3647	13.4963	16.6254
median	13.3696	13.3717	13.3672	13.3635	36.4074	13.3686	13.3775	13.3616	13.3609	16.3014

**Table 4:** Comparison table of optimization results for three-bar truss design.

Cantilever Beam	CAED	ED	PSO	GWO	AOA	DBO	GJO	SCSO	BKA	SABO
worst	259.8050467	259.8050467	259.8050467	259.8050675	259.8500987	259.8050467	259.8050759	259.8050484	259.8050467	259.8243953
best	259.805047	259.8050477	259.8050467	259.8062248	262.649983	259.8050467	259.8120313	259.8052105	259.8050467	260.3863756
std	1.1171 × 10^−7^	3.21343 × 10^−7^	1.15786 × 10^−11^	0.000340549	0.809067725	6.71779 × 10^−13^	0.00247157	4.89464 × 10^−5^	4.96273 × 10^−13^	0.17902373
mean	259.8050467	259.8050469	259.8050467	259.8053782	260.4395624	259.8050467	259.8076083	259.8050817	259.8050467	260.0190289
median	259.8050467	259.8050469	259.8050467	259.8053139	260.2590527	259.8050467	259.8069449	259.8050596	259.8050467	259.9452844

## Data Availability

The data that support the findings of this study are available from the corresponding author upon request. There are no restrictions on data availability.
